# The *Arabidopsis thaliana* Poly(ADP-Ribose) Polymerases 1 and 2 Modify DNA by ADP-Ribosylating Terminal Phosphate Residues

**DOI:** 10.3389/fcell.2020.606596

**Published:** 2020-11-26

**Authors:** Sabira Taipakova, Aigerim Kuanbay, Christine Saint-Pierre, Didier Gasparutto, Yeldar Baiken, Regina Groisman, Alexander A. Ishchenko, Murat Saparbaev, Amangeldy K. Bissenbaev

**Affiliations:** ^1^Department of Molecular Biology and Genetics, Faculty of Biology and Biotechnology, Al-Farabi Kazakh National University, Almaty, Kazakhstan; ^2^Groupe «Mechanisms of DNA Repair and Carcinogenesis», Equipe Labellisée LIGUE 2016, CNRS UMR 9019, Université Paris-Saclay, Villejuif, France; ^3^CEA, CNRS, IRIG/SyMMES-UMR 5819/CREAB, Université Grenoble Alpes, Grenoble, France; ^4^National Laboratory Astana, Nazarbayev University, Nur-Sultan, Kazakhstan; ^5^School of Engineering and Digital Sciences, Nazarbayev University, Nur-Sultan, Kazakhstan

**Keywords:** plant DNA repair, *Arabidopsis thaliana*, DNA strand break, nicotinamide adenine dinucleotide (NAD^+^), poly(ADP-ribose) polymerase (PARP), ADP-ribosylation

## Abstract

Proteins from the poly(ADP-ribose) polymerase (PARP) family, such as PARP1 and PARP2, use NAD^+^ as a substrate to catalyze the synthesis of polymeric chains consisting of ADP-ribose units covalently attached to an acceptor molecule. PARP1 and PARP2 are viewed as DNA damage sensors that, upon binding to strand breaks, poly(ADP-ribosyl)ate themselves and nuclear acceptor proteins. The flowering plant *Arabidopsis thaliana* contains three genes encoding homologs of mammalian PARPs: *atPARP1*, *atPARP2*, and *atPARP3*. Both atPARP1 and atPARP2 contain poly(ADP-ribosyl)ating activity; however, it is unknown whether they could covalently modify DNA by ADP-ribosylating the strand break termini. Here, we report that similar to their mammalian counterparts, the plant atPARP1 and atPARP2 proteins ADP-ribosylate 5′-terminal phosphate residues in duplex DNA oligonucleotides and plasmid containing at least two closely spaced DNA strand breaks. AtPARP1 preferentially catalyzes covalent attachment of ADP-ribose units to the ends of recessed DNA duplexes containing 5′-phosphate, whereas atPARP2 preferentially ADP-ribosylates the nicked and gapped DNA duplexes containing the terminal 5′-phosphate. Similar to their mammalian counterparts, the plant PARP-catalyzed DNA ADP-ribosylation is particularly sensitive to the distance that separates two strand breaks in the same DNA molecule, 1.5 and 1 or 2 turns of helix for atPARP1 and atPARP2, respectively. PAR glycohydrolase (PARG) restored native DNA structure by hydrolyzing the PAR–DNA adducts generated by atPARPs. Biochemical and mass spectrometry analyses of the PAR–DNA adducts showed that atPARPs utilize phosphorylated DNA termini as an alternative to protein acceptor residues to catalyze PAR chain synthesis *via* phosphodiester bond formation between C1′ of ADP-ribose and a phosphate residue of the terminal nucleotide in DNA fragment. Taken together, these data establish the presence of a new type of DNA-modifying activity in *Arabidopsis* PARPs, suggesting a possible role of DNA ADP-ribosylation in DNA damage signaling and repair of terrestrial plants.

## Introduction

Land plants are under constant exposition to a variety of abiotic stresses including UV radiation, droughts, temperature variation, salinity, and other environmental extremes that can extensively damage cellular DNA. In addition to that, plants experience endogenous oxidative stress because of reactive oxygen species (ROS) generated during respiration in mitochondria, photorespiration in chloroplasts, and various biotic stresses. Oxidative damage to DNA caused by ROS is believed to be a major source of genome instability and aging (Cadet and Wagner, 2013). Importantly, direct attack of ROS on DNA abstracts hydrogen from deoxyribose carbons leading to single- and double-strand DNA breaks (SSBs and DSBs, respectively) (Balasubramanian et al., 1998). In addition, DNA strand breaks can be generated indirectly during DNA excision repair of modified bases, replication fork collapse, and topoisomerase action (Pommier et al., 2010). If left undetected and unrepaired, DNA strand breaks have detrimental consequences, such as gross chromosomal rearrangements, persistent genome instability, and cell death. The poly(ADP-ribose) polymerase (PARP) superfamily of proteins, also referred to as the diphtheria toxin-like ADP-ribosyltransferase (ARTD) family, is widespread in eukaryotes and has been identified by homology search in all six major eukaryotic supergroups (Perina et al., 2014). The well-characterized PARP enzymes, mammalian PARP1 and PARP2, catalyze the synthesis of long chains of ADP-ribose (PAR) covalently attached to acceptor proteins using nicotinamide adenine dinucleotide (NAD^+^) as a substrate (Kim et al., 2005; Schreiber et al., 2006; Hottiger et al., 2010). It is agreed that PARP1, PARP2, and PARP3 are sensors of DNA damage that are activated by binding to DNA strand discontinuities. After activation of the catalytic domain, PARPs poly/mono-ADP-ribosylate (PARylate/MARylate) themselves and different nuclear proteins, these in turn regulate the functions of ADP-ribosylated proteins. The ADP-ribose polymer synthesized by PARPs has a complex branched structure, which confers a negative charge and thus stimulates electrostatic repulsion of PARylated proteins from DNA (Tanaka et al., 1984; Satoh et al., 1994). It should be stressed that the PARP-catalyzed covalent protein posttranslational modification is a reversible process since PAR is rapidly degraded by poly(ADP-ribose) glycohydrolase (PARG) which specifically hydrolyze the ribose–ribose bonds in the polymer. Hence, the activation of PARP-catalyzed auto-ADP-ribosylation and modification of nuclear proteins such as histones is one of the common cellular responses to DNA damage (De Murcia and Menissier De Murcia, 1994).

**FIGURE 1:**
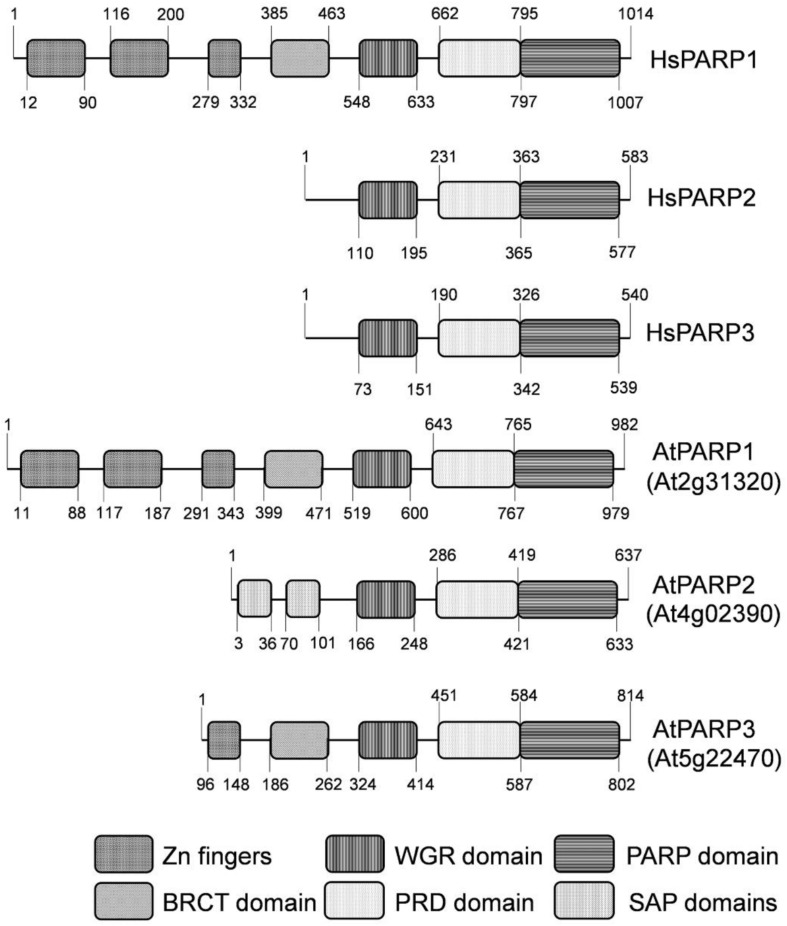
Schematic representation of domain structures of human and *Arabidopsis thaliana* PARP proteins. Zn1, Zn2, and Zn3—three zinc-binding domains; BRCT: BRCA-1 C-terminal domain for phospho-protein binding. WGR—conserved Trp-Gly-Arg motif for putative nucleic acid binding; PRD—PARP regulatory domain; PARP—PARP catalytic domain; SAP—SAF-A/B, Acinus and PIAS motif for putative DNA/RNA binding.

From the past, molecular characterization of DNA repair mechanisms has been mainly focused on bacterial, yeast, and animal cells (Friedberg et al., 2006), with much less attention paid to the mechanisms that maintain the genome stability in plants. The genome of *Arabidopsis thaliana*, a widely used model plant of the dicot group, contains three genes encoding homologs of mammalian poly(ADP-ribose) polymerases (PARPs): *atPARP1*, *atPARP2*, and *atPARP3*. Previously, it was shown that plant PARP1 and PARP2 contain poly(ADP-ribosyl)-transferase activity (Chen et al., 1994; Lepiniec et al., 1995; Babiychuk et al., 1998). Sequence analysis revealed that atPARP1 has a very similar domain architecture to that of human PARP1 (Babiychuk et al., 1998), and it consists of five domains of known functions: three N-terminal zinc finger domains implicated in DNA damage detection, the BRCA1 C-terminus (BRCT) domain for automodification, the WGR domain with the conserved Trp-Gly-Arg (WGR) motif for DNA binding, and the highly conserved catalytic (CAT) domain consisting of two subdomains, ADP-ribosyltransferase catalytic subdomain (ART) and helical subdomain (HD) which is an autoinhibitory domain that blocks productive NAD(+) binding regulating PARP catalytic activity (Eustermann et al., 2015) (Figure 1). The atPARP2 protein contains highly conserved ART subdomain and is structurally most similar to human PARP2 (Figure 1). AtPARP2 has no zinc fingers and BRCT domains like human PARP1, but contains two N-terminal SAF/Acinus/PIAS motif (SAP) domains that confer DNA-binding activity (Aravind and Koonin, 2000; Lamb et al., 2012). Expression of atPARP2 in yeast revealed poly(ADP-ribosyl)ating activity generating mainly short polymers with the size of 10–15 residues; however, longer polymers up to 40 ADP-ribosyl units were also observed (Babiychuk et al., 1998). AtPARP1 and atPARP2 contain a typical histidine-tyrosine-glutamic acid H-Y-E catalytic triad in their conserved ART subdomains, whereas atPARP3 and orthologous plant proteins have an alternative cysteine-valine-glutamic acid (C_653_-V_687_-E_782_ and C_N_-V_N_-E_N_, respectively) triad in their catalytic domain (Citarelli et al., 2010; Stolarek et al., 2015). Noteworthy, a recent study demonstrated that atPARP3 lost NAD(+)-binding capability and poly(ADP-ribose) polymerase activity and may play different biological roles from those of atPARP1 and atPARP2 enzymes in plants (Gu et al., 2019).

The atPARP1 and atPARP2 proteins have nuclear localization and ADP-ribosylate themselves (automodification) and acceptor proteins in the presence of nicked DNA *in vitro* and *in vivo* (Babiychuk et al., 1998; Doucet-Chabeaud et al., 2001; Feng et al., 2015; Liu et al., 2017; Chen et al., 2018). The treatment of *Arabidopsis* with ionizing radiation, zeocin, and DNA cross-linking agent such as cisplatin activates the expression of atPARP1 and atPARP2, but not that of atPARP3 (Doucet-Chabeaud et al., 2001; Boltz et al., 2014; Yuan et al., 2014). In agreement with these observations, *Arabidopsis parp1* and *parp2* single mutant plants exhibit enhanced sensitivity to the alkylating agent methyl methane sulfonate (MMS) and the radiomimetic agent bleomycin (Jia et al., 2013; Boltz et al., 2014; Zhang et al., 2015; Klemm et al., 2017). Interestingly, the *Arabidopsis parp2* single mutants exhibited a stronger decrease in poly(ADP-ribosyl)ation and were more sensitive to bleomycin and mitomycin, as compared with *parp1* single ones (Boltz et al., 2014; Song et al., 2015). Nevertheless, both atPARPs participate in the responses to DNA damage since Arabidopsis *parp1 parp2* double mutants showed increased sensitivity to genotoxic stress, as compared with single *parp* mutants (Jia et al., 2013; Song et al., 2015; Zhang et al., 2015). Additionally, atPARP1 mRNA was induced in *parp2* mutants, and conversely, atPARP2 mRNA was induced in *parp1* mutants (Boltz et al., 2014). Interestingly, atPARP1 and atPARP2 proteins similar to their human counterparts can interact with each other (Song et al., 2015; Liu et al., 2017). Surprisingly, *A. thaliana parp1 parp2 parp3* triple mutants did not exhibit higher sensitivity to DNA damage, as compared with double *parp1 parp2* mutant, suggesting that atPARP3 plays a minor role in DNA damage response and repair in seedlings (Zhang et al., 2015). Nevertheless, mutation in barley PARP3 homolog (HvPARP3) resulted in an altered root growth in response to bleomycin (Stolarek et al., 2015). In contrast to studies on animal models, the plant *Arabidopsis* mutant lines for PARP genes including double and a triple mutant *parp1 parp2 parp3* did not exhibit significant phenotypic abnormalities under normal non-stressed growth conditions (Song et al., 2015; Zhang et al., 2015; Rissel et al., 2017). Nevertheless, plant PARP activity controls cell cycle progression and redox status, suggesting a regulatory function of atPARPs in plant development. In agreement with this, the seed germination is altered in *parp1*, *parp2*, and *parp3* single mutant plants. Under normal non-stressed conditions, parp3 mutant plants germinated faster than the wild type, whereas *parp1* and *parp2* showed reduced germination rates (Pham et al., 2015). Noteworthy, the double *parp1 parp2* mutant of *Arabidopsis* showed more rapid primary and lateral root growth, suggesting that plant PARPs inhibit mitosis and promote cell differentiation (Liu et al., 2017). Based on these observations, it was proposed that PARPs influence plant development only under specific conditions the nature of which requires further investigations (Rissel and Peiter, 2019).

The fact that protein ADP-ribosylation has been increased in the Arabidopsis *parp* triple mutant suggests the presence of supplementary PARP-like enzymes in plants. Indeed, in addition to the canonical PARP proteins, higher plants have a plant-specific family of proteins containing PARP-like domains, called the SRO (Similar to RCD One) proteins. The SRO family possesses a central catalytic PARP domain with an unusual catalytic triad motif (L-H-N) which is flanked by an N-terminal WWE domain [poly(ADP-ribose) binding domain] and a C-terminal RST domain (RCD1-SRO-TAF4—plant-specific protein–protein interaction domain) (Ahlfors et al., 2004; Jaspers et al., 2010). In *Arabidopsis*, the family includes the proteins Radical-induced Cell Death1 (RCD1) and its five homologs SRO1–SRO5 (Belles-Boix et al., 2000; Jaspers et al., 2010). Bioinformatics and biochemical data suggest that the SRO proteins do not have PARP activity; however, mutant analyses have shown that these proteins play a significant role in stress response (Ahlfors et al., 2004; Jaspers et al., 2009; Teotia and Lamb, 2009; Teotia and Lamb, 2011).

At variance to mammals, plants possess two genes atPARG1 (At2g31870) and atPARG2 (At2g31865) encoding for the poly(ADP-ribose) glycohydrolase (Zhang et al., 2015). Under the same conditions, atPARG1 plays an essential role and atPARG2 a minor one. Interestingly, the atPARG1 deficiency results in more DNA damage and enhanced cell death in plants after bleomycin treatment, than the lack of AtPARPs, possibly due to a high toxicity of free poly(ADP-ribose) polymer (Zhang et al., 2015).

Recent studies have demonstrated a new phenomenon of postreplicative DNA ADP-ribosylation of strand break termini in synthetic duplex DNA oligonucleotides; this reaction is catalyzed by mammalian PARP1, PARP2, and PARP3 (Talhaoui et al., 2016; Munnur and Ahel, 2017; Belousova et al., 2018; Zarkovic et al., 2018). PARP1 and PARP2 catalyze the covalent addition of ADP-ribose units to 5′- and 3′-terminal phosphates and to 2′-OH termini of modified nucleotides at DNA strand breaks, producing covalent PAR–DNA adducts (Talhaoui et al., 2016). PARP1 preferentially ADP-ribosylates DNA strand break termini containing terminal phosphates or 2′-OH group in gapped, recessed DNA duplexes, whereas PARP2 preferentially acts on 5′-terminal phosphates at DSB termini of nicked DNA (Talhaoui et al., 2016; Zarkovic et al., 2018). Also, PARP3 can effectively generate mono-(ADP-ribosyl)ated DNA (MAR–DNA) in which ADP-ribose moiety is covalently linked to 5′-terminal phosphate residues at DSB and SSB in DNA substrate (Munnur and Ahel, 2017; Belousova et al., 2018), thus sharing its substrate specificity with PARP2.

Here, we examined the interactions of plant PARP proteins with various DNA substrates using *in vitro* approaches. Our results reveal that both purified *Arabidopsis* atPARP1 and atPARP2 proteins can covalently modify DNA oligonucleotide duplexes by the addition of multiple poly(ADP-ribose) units to 3′ and 5′ extremities of DNA strand breaks. The atPARP-catalyzed covalent DNA ADP-ribosylation is reversible since PARG can efficiently remove the PAR polymer from DNA and restore initial DNA structure. The mechanistic characteristics and possible functional role of the new activity of plant atPARPs are discussed.

## Materials and Methods

### Bacterial Strains, Plasmids, and Reagents

Cell culture media were from Invitrogen (Life Technologies SAS, Saint Aubin, France). The *Escherichia coli* Rosetta 2(DE3) cells, used for the recombinant protein expression, were from Novagen-EMD4 Biosciences (Merck Chemicals, Nottingham, United Kingdom). Restriction enzymes, T4 DNA ligase, RPROTKSOL-RO: recombinant PCR grade Proteinase K were from Roche (Basel, Swiss), deoxyribonuclease I from bovine pancreas (DNAse I) was from ThermoFisher Scientific (Lithuania), and calf-intestinal alkaline phosphatase (CIP) was from New England Biolabs France (Evry, France). Snake venom phosphodiesterase 1 from *Crotalus adamanteus* (SVPDE1) was from Worthington (Biochemical Corporation). The purified human Nudix (nucleoside diphosphate-linked moiety X)-type motif 16 (NUDT16) protein was prepared as described (Palazzo et al., 2015). Bovine PARG was from Trevigen (Gaithersburg, United States). Bleomycin was from Sanofi-Aventis (France).

### Oligonucleotides

Sequences of the oligonucleotides and their duplexes used in the present work are shown in Supplementary Table S1. All oligonucleotides were purchased from Eurogentec (Seraing, Belgium) including modified oligonucleotides. Prior to enzymatic assays, the oligonucleotides were labeled either at the 5′ end using T4 polynucleotide kinase (New England Biolabs-OZYME, France) in the presence of [γ-^32^P]ATP (3,000 Ci mmol^–1^) (PerkinElmer) or at the 3′ end by means of terminal deoxynucleotidyl transferase (New England Biolabs) in the presence of [α-^32^P]-3′-dATP (cordycepin 5′-triphosphate, 5,000 Ci mmol^–1^; PerkinElmer) according to the manufacturer’s protocol. Cold ATP at 1 mM was added to phosphorylate the remaining non-labeled oligonucleotides. After the reactions, radioactively labeled oligonucleotides were desalted on a Sephadex G-25 column equilibrated with water and then annealed with a corresponding complementary strand for 3 min at 65°C in the buffer containing 20 mM HEPES-KOH (pH 7.6) and 50 mM KCl. In addition, the radioactive labeling of DNA and proteins was performed using [adenylate-^32^P] NAD^+^ (800 Ci mmol^–1^) (PerkinElmer) in the presence of atPARPs, oligonucleotides, and 1 mM cold NAD^+^. To prepare a 5′-[^32^P]labeled linearized nicked plasmid DNA substrate, 50 μM pML2 plasmid (same as pBluescript but contains insertion of a unique *Pml*I site) was linearized with 30 U of *Pml*I for 1 h at 37°C in 1 × CutSmart buffer (New England Biolabs, France), then nicked with 15 U of Nb.BsmI for 1 h at 65°C. The ^32^P label was introduced by reannealing of the linearized pML2 with 5′-[^32^P]-labeled ExoA d(GTGGTTGTAAAACCTCAGCCAG) oligonucleotide corresponding to the 22-nt fragment spanning the region between the 5′ end of DSB and Nb.BsmI-induced nick. The nicked, gapped, or recessed DNA duplexes ExoA•RexT^nick/gap/rec^ composed of RexT d(GGAATTCCCCGCGCCAAATTTCTCTAAGTCTCCGCGCC AC), ExoA d(GTGGCGCGGAGACTTAGAGAA), and either 5P-Exo19 d(pATTTGGCGCGGGGAATTCC) or 5P-Exo18, d(pTTTGGCGCGGGGAATTCC), where 5P is a 5′-terminal phosphate (Supplementary Table S1), were mostly used to quantify PARylation of DNA ends by atPARPs.

### Plant Material, Cell-Free Extracts, and Genomic DNA Extraction

The *A. thaliana* wild-type (WT) Col-0 strain and mutant lines, harboring T-DNA insertions in the atPARP genes, were obtained from the Arabidopsis Biological Resource Center^1^. For all plants, seeds were sown on 1/2 Murashige–Skoog (MS) agar plates containing 1% sucrose and 1% agar, stratified for 48 h at 4°C and grown under long day conditions at 22°C under 16 h light/8 h dark cycles. They were collected at 18 days and transplanted to soil for seed harvest. The *A. thaliana* wild-type Col-0 strain and mutant line seeds were stratified and then grown for 14 days on MS agar plate. Two-week-old *Arabidopsis* seedlings were treated with 50 μg•ml^–1^ bleomycin (MS plates with plants were covered with 5 ml PBS containing the drug) and plants were collected after 24 h.

### Cell-Free Extract Preparation

Extracts were prepared from 100 mg fresh untreated or treated with bleomycin 14-day-old seedlings (whole plant) in extraction buffer (20 mM HEPES-KOH, pH 7.6, 50 mM KCl, 5 mM MgCl_2_, 1 mM DTT, 10% glycerol, 0.1% NP-40 and protease inhibitor cocktail at 1:100).

### Genomic DNA Preparation

The plant genomic DNA (gDNA) was extracted from 100 mg fresh untreated or treated with bleomycin 14-day-old seedlings (whole plant) in liquid nitrogen using the CTAB method. Extracted gDNA were purified further by RNase A and proteinase K treatments, followed by phenol/chloroform extraction and ethanol precipitation of DNA.

### RNA Analysis and cDNA Synthesis

Total RNA was extracted from 100 mg of fresh leaf tissues of *A. thaliana* in liquid nitrogen using the TRIzol reagent (Invitrogen) according to the manufacturer’s instructions. Intact, high-quality RNA samples were confirmed by the presence of two bright 28S and 18S rRNA bands in ethidium bromide-stained agarose gels visualized under UV light. Five micrograms of DNA-free total RNA was converted into single-stranded DNA using a mix of oligo-dT_20_ primers and the First Strand cDNA Synthesis Kit (Thermo Scientific). PCR was performed using 2 μl of a 20-fold dilution of cDNA, 15 pmol of each primer, and 1 U of Taq polymerase in a 25-μl reaction volume. To generate the cDNA for full-length atPARP1 (corresponds to the gene At2g31320) and atPARP2 (corresponds to the gene At4g02390), the coding sequences were PCR-amplified using primers atPARP1*Nde*I_F/atPARP1*Bam*HI_R for atPARP1 and atPARP2*Nde*I_F/atPARP2*Bam*HI_R for atPARP2 (Table 1). The PCR fragments of atPARP1 and atPARP2 were cloned into the pBluescriptII SK(+) vector at *Nde*I/*Bam*HI restriction sites, respectively, using the Rapid DNA ligation kit (Thermo Scientific). Colonies of transformed *E. coli* DH5α cells carrying plasmids with an insert were screened by *lacZ* complementation, and the plasmid DNA was isolated with the GeneJET Plasmid Miniprep kit (Thermo Scientific). The DNA inserts were sequenced in both directions with M13 forward and reverse primers.

**TABLE 1:** List of PCR primers used for cloning and site-directed mutagenesis.

atPARP1*Nde*I_F	CAGCCATATGGCAAGCCCTCATAAGC
atPARP1*Bam*HI_R	AGGCGGATCCTTAGCGTTTGTGTTTAAAGC
atPARP2*Nde*I_F	CAGCCATATGGCAAACAAGCTGAAGG
atPARP2*Bam*HI_R	AGGCGGATCCTTAATGTTTGTAGTTG
atPARP1_E960K_F	CGAACTGATGTATAACAAATATATTGTATATGATAC
atPARP1_E960K_R	GTATCATATACAATATATTTGTTATACATCAGTTCG
atPARP1_E960Q_F	CGAACTGATGTATAACCAATATATTGTATATGATAC
atPARP1_E960Q_R	GTATCATATACAATATATTGGTTATACATCAGTTCG
atPARP2_E614K_F	GCATGCTGCTGTATAACAAATATATTGTTTATAAC
atPARP2_E614K_R	GTTATAAACAATATATTTGTTATACAGCAGCATGC

### Expression and Purification of atPARP1 and atPARP2 Proteins

The cDNA fragments encoding atPARP1 and atPARP2 were subcloned into the *Nde*I and *Bam*HI restriction sites of the pET28c vector. The resulting plasmids pET28c-atPARP1 and pET28c-atPARP2 can express the recombinant proteins containing an N-terminal His-tag in an *E. coli* (DE3) strain. The following mutants atPARP1^E960K^, atPARP1^E960Q^, and atPARP2^E614K^ were constructed using the QuikChange site-directed mutagenesis kit (Stratagene) and the oligonucleotide primers are shown in Table 1. The WT and mutant atPARP proteins were purified from *E. coli* Rosetta 2 (DE3) strain (Merck). Briefly, the transformed *E. coli* cells were grown to OD_600_ ∼ 0.6 at 37°C in LB medium and then induced by incubating with 50 μM isopropyl β-D-1-thiogalactopyranoside overnight at 25°C. Owing to strong expression in the Rosetta strain, it was possible to purify the atPARP1 and atPARP2 proteins to near homogeneity using only two chromatographic steps. All purification procedures were carried out at 4°C. Bacteria were harvested by centrifugation, and cell pellets were lysed using a French press at 18,000 psi in a buffer containing 50 mM Tris-HCl pH 8.0, 100 mM NaCl, 1 mM EDTA, 5% glycerol, 1 mM DTT, and 0.5% NP-40 supplemented with Complete Protease Inhibitor Cocktail (Roche Diagnostics, Switzerland). The lysates were cleared by centrifugation at 40,000 × *g* for 60 min at 4°C, and the resulting supernatant was adjusted to 500 mM NaCl and 20 mM imidazole and loaded onto a HiTrap Chelating HP column (GE Healthcare) charged with Ni^2+^. The eluted fractions containing the recombinant proteins were pooled and loaded onto a 1-ml HiTrap-Heparin column (GE Healthcare). The bound proteins were eluted in a 50–1,500-mM NaCl gradient. The homogeneity of the purified proteins were assessed by using the SDS-PAGE method (Supplementary Figure S1). The purified protein samples were stored at -20°C in 50% glycerol.

### Preparation of Anti-atPARP2 Antibodies and Western Blotting

The anti-atPARP2 polyclonal antibodies were raised against the full-length recombinant His-tagged *Arabidopsis* atPARP2 protein. Approximately 1 mg of the purified recombinant atPARP2 protein was mixed with Freund’s complete adjuvant and injected into rabbits. Three additional injections were made at 2-week intervals. One week after the last injection, the blood was collected and the immune serum was affinity purified using protein A agarose fast flow resin (Sigma). The purified rabbit anti-atPARP2 polyclonal antibodies were used as primary antibodies, and the horseradish peroxidase-conjugated goat anti-rabbit IgG was used as a secondary antibody. Plant cell-free extracts (∼12 μg of protein) were separated in a 10% SDS-polyacrylamide gel and then electroblotted onto a polyvinyl difluoride membrane (Pierce) using a Bio-Rad Mini-transblot cell according to the manufacturer’s instructions. After the transfer of proteins, the membrane was gently shaken in blocking solution containing 5% milk and 0.1% Tween-20 in 1 × TBS (Tris-buffered saline: 50 mM Tris-HCl pH 7.5, 20 mM NaCl) for 1 h at room temperature. After removing the blocking solution, the membrane was incubated in 10 ml of the affinity-purified anti-atPARP2 antibodies (1:30,000 dilution in the blocking solution with 0.1% Tween-20) overnight at 4°C. The membrane was washed five times in 10 ml of the wash buffer (1 × TBS supplemented with 0.1% Tween-20), for 5 min each time. After washing, the membrane was incubated with the secondary antibody (1:60,000 dilution in the blocking solution with 0.1% Tween-20) in 10 ml for 1 h at room temperature. Then the membrane washed five times in 10 ml of the wash buffer, for 5 min each time. The working substrate solution was prepared by mixing an equal volume of peroxide solution and luminal/enhancer solution and used at 0.1 ml cm^–2^ per blot area. The membrane was incubated in the working solution for 2 min in the dark and exposed to Kodak X-Omat film.

### Activity Assay for Poly(ADP-Ribose) Polymerase

The standard DNA PARylation assay (10 μl) was performed by incubating 20 nM [^32^P]-labeled oligonucleotide, 250 nM atPARP1 or atPARP2, and 1 mM NAD^+^, in ADPR buffer [20 mM HEPES-KOH pH 7.6, 50 mM KCl, 5 mM MgCl_2_, 1 mM DTT, and μg•ml^–1^ bovine serum albumin (BSA)], for 30 min at 37°C, unless otherwise stated. After the reaction, the samples were incubated in the presence of 50 μg•ml^–1^ proteinase K and 0.15% SDS for 30 min at 50°C followed by incubation for 3 min at 95°C. The samples were desalted on a Sephadex G-25 column (Amersham Biosciences) equilibrated in 7.5 M urea, and then the products were analyzed by electrophoresis in the denaturing 20% (w/v) polyacrylamide gel (PAGE, 7 M urea, 0.5 × TBE, 42°C). A wet gel was wrapped in a plastic drape, then exposed to a Storage Fuji FLA-3000 Phosphor Screen, which was then scanned using Typhoon FLA 9500, and digital images were obtained and quantified using FUJI Image Gauge V3.12 software.

### Hydrolysis of the ADP-Ribosylated DNA Adducts by PARG and DNA-Modifying Enzymes

The hydrolysis of PAR–DNA and MAR–DNA adducts with PARG was performed after denaturing of the atPARP proteins by heating a sample for 20 min at 80°C, then 50 pg•μl^–1^ PARG was added to the reaction mixtures, and samples were incubated for 30 min at 37°C. The reaction products were analyzed as described above. The dephosphorylation assay with CIP was performed using 10 U of the enzyme in the CIP buffer (provided by New England Biolabs) for 1 h at 37°C. The hydrolysis of the PAR–DNA polymers by SVPDE1 was performed in two steps: first, incubation of 20 nM DNA substrate with 250 nM atPARP2 and 1 mM NAD^+^ in the PARP buffer (see above) for 30 min at 37°C, and then the second step was incubation with 100 mU SVPDE1 in the reaction mixture supplemented with 10 mM MgCl_2_ and 75 mM Tris-HCl pH 8.9, for 1 h at 37°C. In addition, the SVPDE1 reaction products were treated with 10 U CIP in the CIP buffer for 40 min at 37°C, unless otherwise stated. The reaction products were analyzed as described above. Hydrolysis of the PAR–DNA polymer by the Nudix hydrolase was performed using 2–20 μM NUDT16 in the DNA PARylation assay buffer supplemented with 10 mM MgCl_2_ for 18 h at 30°C, unless otherwise stated.

### Identification of the ADP-Ribosylated DNA Adducts by Matrix-Assisted Laser Desorption Ionization Time-of-Flight Mass Spectrometry

Mass spectrometry measurements were performed as described previously (Talhaoui et al., 2016). Briefly, 5 μM of cold non-radioactive 5′-phosphorylated 30-mer nicked duplex oligonucleotide [referred to here as p10•RT-A^Nick^ or S18 and composed of a 30-mer (RT-A) template strand and two 5′-phosphorylated complementary strands: 10-mer (p10) and 20-mer (pT19)] (Supplementary Table S1) was incubated with 2.5 μM atPARP2 in the presence of 1 mM NAD^+^ at 37°C for 1 h. After incubation, the reaction was stopped by heating the samples for 20 min at 80°C. Then, the reaction products were precipitated with 2% lithium perchlorate in acetone, desalted, and used for the matrix-assisted laser desorption ionization time-of-flight (MALDI-TOF) mass spectrometry (MS) measurements. MALDI-TOF mass spectra were obtained in the negative mode on a Microflex mass spectrometer (Bruker, Wissembourg, France), equipped with a 337-nm nitrogen laser and pulsed delay source extraction. The matrix was prepared by dissolving 3-hydroxypicolinic acid in 10 mM ammonium citrate buffer and a small amount of Dowex-50W 50 × 8-200 cation exchange resin (Sigma). The matrix (1 μl) was added to the sample (1 μl) on the target plate and allowed to dry. The spectra were calibrated using reference oligonucleotides of known masses.

### Analysis of the Efficiency of atPARP2-Catalyzed Auto- and DNA ADP-Ribosylation

The efficiency of atPARP2-catalyzed auto- and DNA ADP-ribosylation was measured using a cold ExoA•RexT^Nick^ duplex phosphorylated at the 5′ end of the nick and with or without a phosphate at the 5′ DSB terminus. The assay was performed in the ADPR buffer without BSA. One micromolar atPARP2 was incubated in the presence of 10 μM oligonucleotide duplex and 1 μM [adenylate-^32^P]NAD^+^ for 30 min at 37°C. The reactions were terminated by the addition of the stop solution (7.5 M urea, 0.33% SDS, 10 mM EDTA, and 0.25% bromophenol blue) at 1:1 (v/v) and heating at 95°C for 10 s, after which the products of the reactions were analyzed on denaturing PAGE as described above.

### Analysis of Plant Genomic DNA for the Presence of PAR–DNA Adducts

Isolation of gDNA was described above in the *Plant Material, Cell-Free Extracts, and Genomic DNA Extraction* section. To examine the presence of ADP-ribosylated DNA adducts in plant, 1,000 ng of gDNA extracted from the control and treated 14-day-old seedlings (whole plant) were dot blotted on a Hybond N^+^ nylon membrane (GE Healthcare). DNA and PAR were fixed to the membrane by heating at 80°C for 2 h and then analyzed with the mouse monoclonal anti-poly(ADP-ribose) antibody 10H (1:2,000, Enzo Life Sciences Inc., United States) and the rabbit monoclonal *anti*-*pan*-*ADP*-*ribose* binding reagent MABE1016 (1:2,000, Millipore, United States). Immunodetection of poly(ADP-ribose) on the blot strips has been performed by using the ECL method, followed by a scan with Amersham^®^ Imager 600 instrument (GE Healthcare) and by short exposure to blue-light-sensitive autoradiography film.

## Results

### Plant atPARP1 and atPARP2 Modify DNA Oligonucleotide Duplexes in the Presence of NAD^+^

**FIGURE 2:**
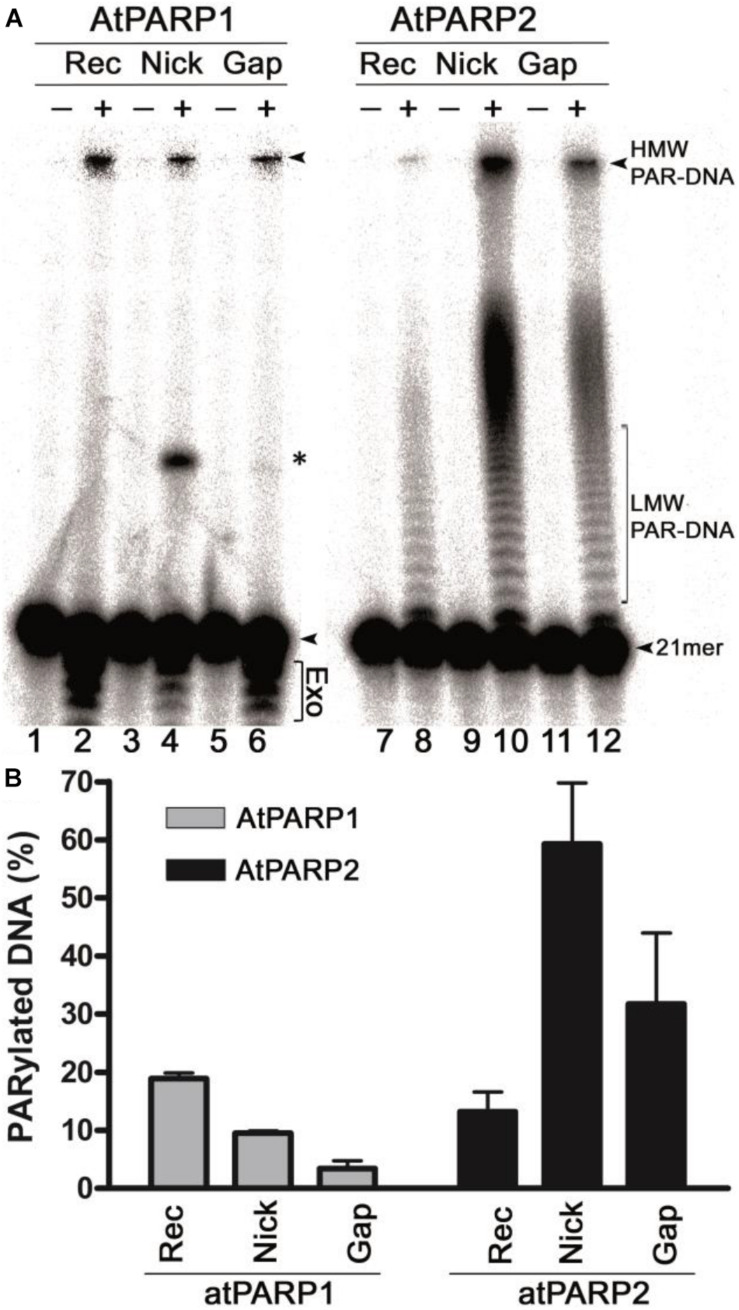
AtPARP-catalyzed formation of radioactively labeled high-molecular-weight (HMW) DNA products. **(A)** Denaturing PAGE analysis of atPARP1- and atPARP2-generated HMW products after incubation with 5′-[^32^P]-labeled 40-mer nicked, gapped, and recessed DNA duplexes. Arrows indicate the 21-mer free oligonucleotide, HMW and LMW PAR–DNA products. Asterisk indicates a nonspecific ligation product generated by NAD^+^-dependent *E. coli* DNA ligase A. “Exo” indicates exonuclease degradation products of 21-mer fragment. For details, see section “Materials and Methods.” **(B)** Graphic representation of the formation of HMW and LMW products by atPARPs when acting upon gapped, nicked, and recessed DNA duplexes. Each bar represents atPARP activity as mean ± SD from three independent experiments.

In our previous work, we have demonstrated that *in vitro* mammalian PARP1 and PARP2 proteins can poly(ADP-ribosyl)ate duplex oligonucleotides containing multiple closely spaced DNA strand breaks and phosphorylated termini. *A. thaliana* atPARP1 and atPARP2 proteins share homology with mammalian PARP1 and PARP2 proteins, respectively, suggesting that the plant proteins might also exhibit DNA modification activities. To verify this, we examined the biochemical activities of the purified atPARP1 and atPARP2 proteins using DNA substrates containing more than two strand breaks to mimic clustered DNA damage and repair intermediates: ExoA•RexT^Nick^ (referred to as S13) and ExoA•RexT^gap^ (referred to as S10), which are 40-mer oligonucleotide duplexes containing a nick and one-nucleotide gap, respectively, composed of a 40-mer (RexT, also referred to as S1) template strand and two complementary strands: 21-mer (ExoA) and phosphorylated 18-mer (5′pExo18) or 19-mer (5′pExo19) strands (Supplementary Table S1). In addition, we prepared ExoA•RexT^rec^ (referred to as S3, S5, and S6) and Exo20•RexT^rec^ (referred to as S4), which are recessed duplexes with a 5′ single-stranded tail, composed of RexT and ExoA or Exo20, respectively. In ExoA•RexT^rec^ duplex, either ExoA or RexT was [^32^P]-labeled at the 5′ end, and in Exo20•RexT^rec^, Exo20 was [^32^P]-labeled at the 3′ end. The 5′-[^32^P] labeled oligonucleotide duplexes were incubated with the atPARP proteins in the presence of 1 mM NAD^+^; the reactions were stopped by adding 0.15% SDS and 50 μg•ml^–1^ proteinase K and incubating for 30 min at 55°C. After this, the samples were desalted and then heat treated (5 min at 95°C) in a gel loading buffer, and the products were separated by electrophoresis on a denaturing polyacrylamide gel. Analysis of the reaction products revealed that 3–60% of the [^32^P]-labeled oligonucleotides are converted to slowly migrating DNA products which run on the gel above the non-modified 21-mer fragment (Figure 2A, lanes 2, 4, 6, 8, 10, and 12), suggesting that the PAR polymer synthesized by atPARPs generated a complex with DNA. Importantly, these slowly migrating PAR–DNA products were resistant to proteinase K, SDS, and heat treatment pointing to a possible covalent nature of the atPARP-induced DNA modifications. Noteworthy, atPARP2 modifies DNA more efficiently as compared with atPARP1 (Figures 2A,B). Particularly, atPARP1 generated mainly high-molecular-weight (HMW) PAR–DNA products, which were unable to enter the gel (Figure 2A, lanes 2, 4, and 6), whereas atPARP2 produced, in addition to HMW, low-molecular-weight (LMW) PAR–DNA products which were able to enter the gel and migrated as a ladder of distinct DNA fragments above the free 21-mer fragment (lanes 8, 10, and 12). As shown in Figure 2B, the relative efficiency levels of the atPARP1- and atPARP2-catalyzed formation of PAR–DNA products were strongly dependent on DNA duplex structures. AtPARP1 preferentially modifies the recessed duplex ExoA•RexT^rec^ (S5) (20% of HMW PAR–DNA products) and to a lesser extent the gapped and nicked DNA duplexes (3 and 10% of HMW PAR–DNA products, respectively), whereas atPARP2 prefers gapped and nicked duplexes (30 and 60% of LMW and HMW PAR–DNA products, respectively) as compared with a recessed DNA (14% of PAR–DNA products). It should be noted that atPARP1, but not atPARP2, induces limited non-specific 3′→5′ exonuclease degradation of the 21-mer fragment (Figure 2A, lanes 2, 4, and 6), suggesting a non-specific DNA exonuclease contamination in recombinant atPARP1 preparation. Furthermore, the incubation of atPARP1 with ExoA•RexT^Nick^ (S13) and, to a much lesser extent, with ExoA•RexT^gap^ (S10) produces a discrete band migrating at the position of the 40-mer fragment (lanes 4 and 6), suggesting the presence of a DNA ligase activity in the purified plant protein. These observations suggest that the recombinant atPARP1 protein, despite extensive purifications, is contaminated by the host NAD^+^-dependent *E. coli* DNA ligase A and non-specific DNA exonucleases.

**FIGURE 3:**
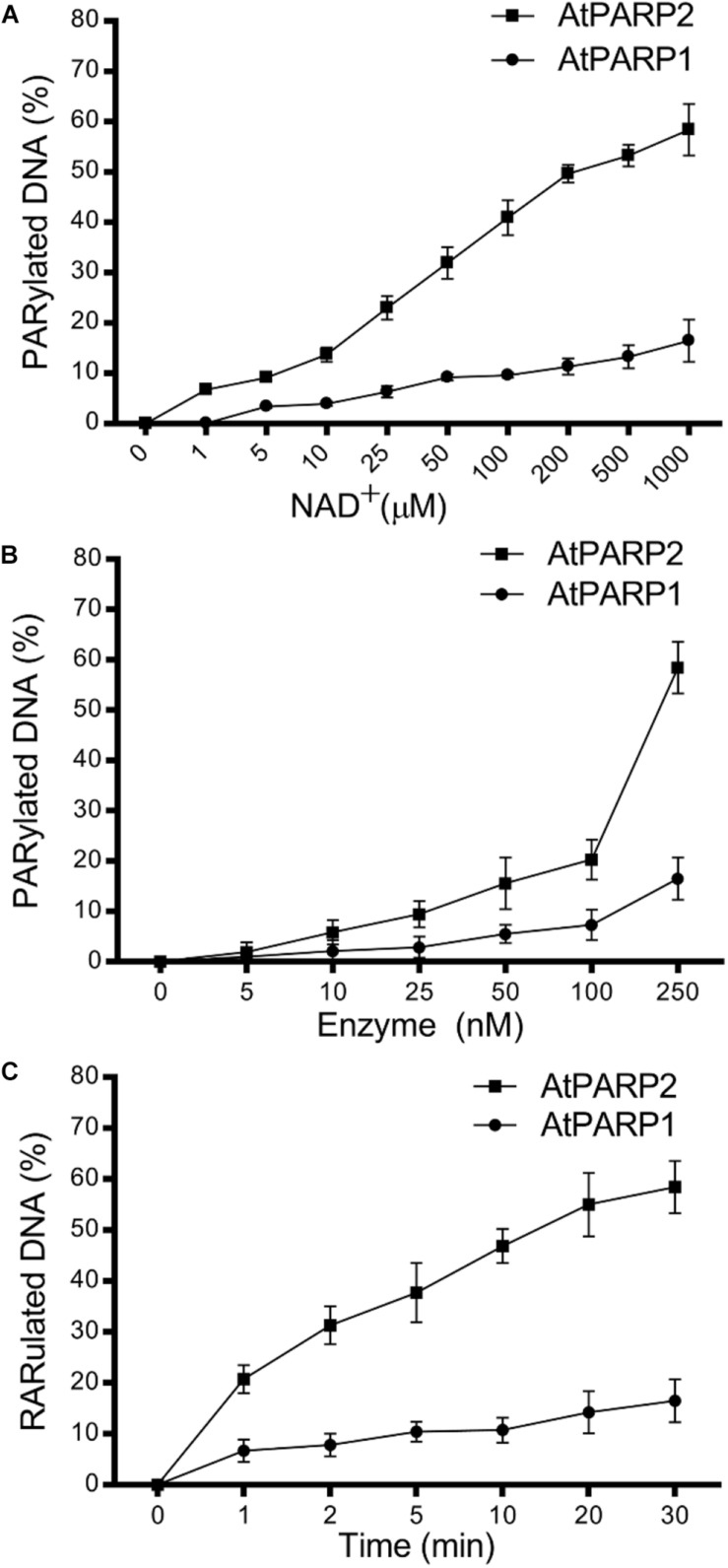
Dependence of the atPARP-catalyzed PAR–DNA formation on reaction conditions; 250 nM atPARP1 and atPARP2 incubated with 20 nM DNA duplex for 30 min at 37°C. **(A)** atPARP1- and atPARP2-catalyzed DNA PARylation in the presence of varying concentrations of NAD^+^ (1–1,000 μM). **(B)** The dependence of DNA PARylation on atPARP protein concentrations (0–250 nM). **(C)** Time dependence of DNA PARylation by atPARPs (0–30 min). For details, see section “Materials and Methods.”

Next, we examined time, NAD^+^, and protein concentration dependence of the PAR–DNA product formation by the atPARP proteins. For this, we incubated atPARP1 and atPARP2 with their preferred substrates 5′-[^32^P]-labeled ExoA•RexT^rec^ (S5) and ExoA•RexT^Nick^ (S13), respectively, under varying concentrations of NAD^+^ and protein. For atPARP1, the PAR–DNA products were not formed in the absence or at very low concentrations of NAD^+^ (0–10 μM) and protein (5–50 nM) (Figures 3A,B), but the level of DNA modification steadily increased at higher concentrations of NAD^+^ (from 25 μM to 1 mM) and protein (100–250 nM) (Figures 3A,B and Supplementary Figure S2). Noteworthy, the DNA PARylation activity of atPARP1 quickly reached the plateau level after only 1 min incubation and increased very little following 30 min incubation (Figure 3C). Similarly for atPARP2, the DNA PARylation was very low or absent at low concentrations of NAD^+^ (0–25 μM) (Figure 3A) and protein (5–10 nM) (Figure 3B), but the activity steadily increased at higher concentrations of NAD^+^ (0.1–1 mM) and protein (25–250 nM) (Figure 3 and Supplementary Figure S2). Noteworthy, when the protein concentration was below 250 nM, atPARP2 was not able to generate the HMW PAR–DNA products, but only LMW products (Supplementary Figure S2). It should be noted that atPARP2, but not atPARP1, generated LMW PAR–DNA products that migrate as a ladder of distinct DNA fragments above the 21-mer free oligonucleotide which becomes a smear at the distance of 1/3 from the start of the gel (Supplementary Figure S2). The appearance of the DNA ladder implies distributive synthesis of PAR polymer by atPARP2, whereas the formation of HMW PAR–DNA fragments by both atPARP enzymes suggests a high processivity of the synthesis of PAR polymer by plant PARP enzymes (Supplementary Figure S2). Taken together, these results suggest that the plant PARPs, similar to their mammalian counterparts, can synthesize long PAR polymers covalently attached to DNA.

### Characterization of the DNA Substrate Specificity of atPARP-Catalyzed PARylation

**FIGURE 4:**
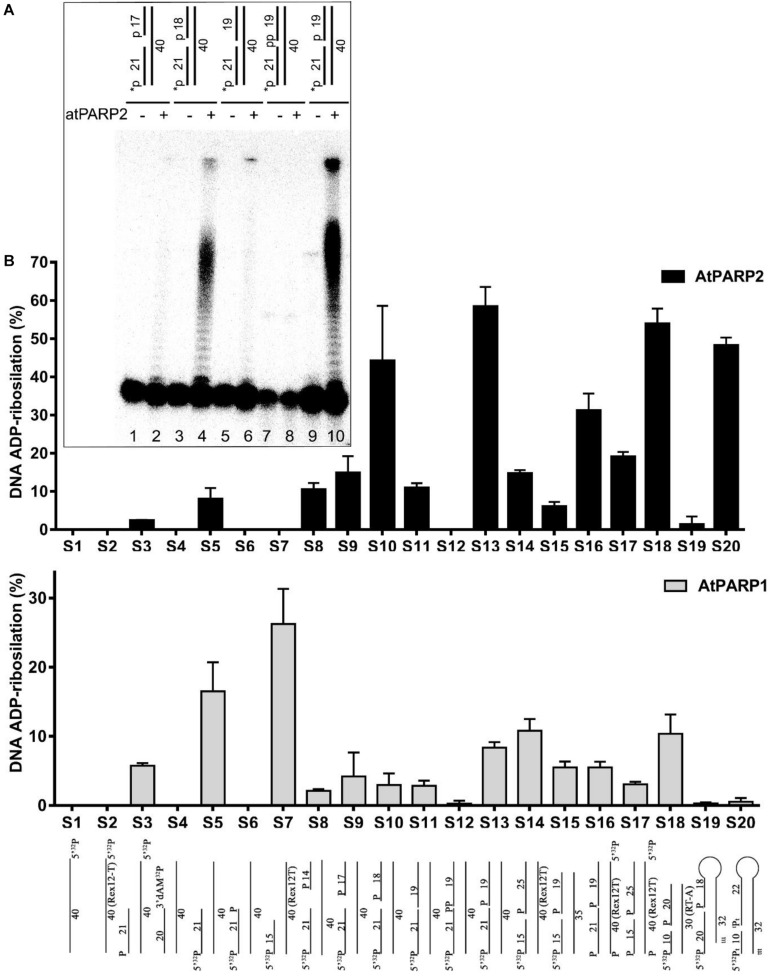
Effect of the DNA structure and nature of termini on the atPARP1- and atPARP2-catalyzed formation of PAR–DNA adducts; 250 nM atPARP proteins were incubated with 20 nM [^32^P]-labeled oligonucleotide in the presence of 1 mM NAD^+^ for 30 min at 37°C. The products of the reaction were separated using denaturing PAGE and the relative amounts of the PAR–DNA products were measured. **(A)** Denaturing gel showing the influence of terminal DNA phosphate residues on atPARP2-catalyzed DNA PARylation. **(B)** Graphic representation of the effects of various DNA structures on atPARP1- and atPARP2-catalyzed DNA PARylation. The data on PARP-catalyzed formation of PAR–DNA products are presented as mean ± SD from three independent experiments. For details, see section “Materials and Methods.”

Next, we assessed in more detail the influence of the different DNA structures [nick, gap, recessed duplexes and single-stranded (ss) DNA] and nature of DNA termini on the atPARP-catalyzed formation of PAR–DNA adducts. For this purpose, we incubated [^32^P]-labeled DNA oligonucleotides of various configurations and terminus structures in the presence of atPARPs and NAD^+^. After incubation, the reaction products were analyzed on the denaturing PAGE and the formation of the PAR–DNA adducts was quantified (Figure 4A). Quantification of the results shown in Figure 4B revealed that (i) overall, atPARP2 was more active as compared with atPARP1 on the majority of DNA substrates tested; (ii) atPARP1 was more active on the recessed DNA duplex (^32^pExo15•Rex12T^rec^ or S7) which contains the 15-mer fragment, as compared with ^32^pExoA•RexT^rec^ duplex (S5) with longer 21-mer strand; and (iii) the substrate preference of atPARP2 was opposite to that of atPARP1, since atPARP2 was active on the latter DNA substrate (^32^pExoA•RexT^rec^ or S5), but lost its activity on the former one (^32^pExo15•Rex12T^rec^ or S7). Interestingly, the presence of two 5′-terminal phosphates in ExoA•RexT^nick/gap^ duplexes (S10 and S13) was necessary for a more efficient DNA PARylation, whereas the presence of the 3′-terminal phosphate and cordycepin (3′-dAMP) in S12 and S4, respectively, strongly inhibited DNA modification (Figure 4). Taken together, these results suggest that the substrate specificities of plant atPARP1 and atPARP2 proteins resemble to their mammalian counterparts PARP1 and PARP2, respectively.

### Construction and Characterization of Catalytic Site atPARP1^E960K, E960Q^ and atPARP2^E614K^ Mutants

The catalytic domain of PARP1, also referred to as ADP-ribosyltransferase (ART) domain, is highly conserved in all PARP family members and shares structural similarity with the plant ADP-ribosylating enzymes. Active mammalian PARPs share a conserved histidine-tyrosine-glutamic acid (H-Y-E) triad (PARP signature) in their catalytic domains (Hassa and Hottiger, 2008). This evolutionary conserved “H-Y-E” triad is essential for the positioning of NAD^+^ during ADP-ribosylation: in PARP1, H862 and Y896 participate in the binding of NAD^+^, while E988 is critical for catalysis and substrate positioning. Y896 stacks with the nicotinamide ring (Steffen et al., 2013), H862 binds to the 2′-OH of NAD^+^ adenine-ribose, and E988 makes a hydrogen bond with the 2′-OH of the nicotinamide-ribose and polarizes the NAD^+^ molecule for nucleophilic attack (Ruf et al., 1998). Alignment of amino acid sequences of ART domains of PARPs revealed a significant homology between human and plant enzymes: PARP1 shared 49.6 and 45.6% homology with atPARP1 and atPARP2, respectively. Noteworthy, human and plant PARPs shared conserved catalytic triad H-Y-E: the catalytic triad of human PARP1 H862-Y896-E988 corresponds to that of atPARP1 consisting of H833-Y867-E960 and atPARP2 consisting of H486-Y520-E614 residues.

**FIGURE 5:**
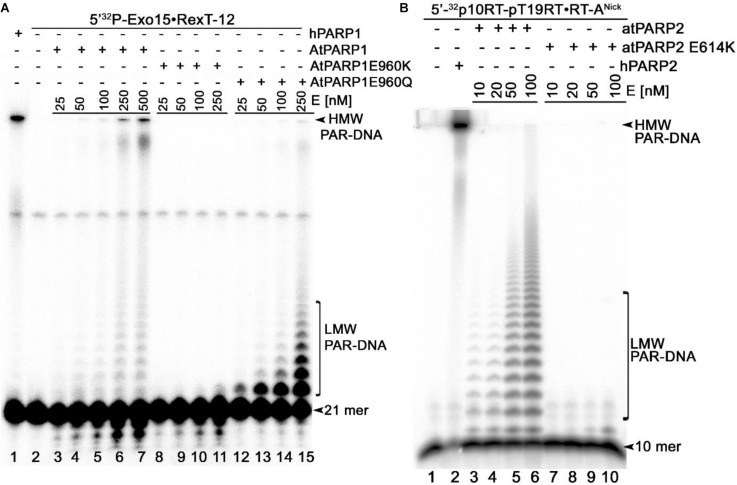
Denaturing PAGE analysis of the PAR–DNA adducts generated by atPARP mutants; 25–500 nM atPARP1 variants and human PARP1 were incubated with 20 nM 5′-[^32^P]-labeled Exo15•Rex12T^Rec^ duplex (S7) and 1 mM NAD^+^ at 37°C for 30 min. On the other hand, 10–100 nM atPARP2 variants and human PARP2 were incubated with 20 nM 5′-[^32^P]-labeled 10RT pT19RT•RT-A^Nick^ duplex (S18) and 1 mM NAD^+^ at 37°C for 30 min. **(A)** Analysis of the PAR-DNA products generated by atPARP1-WT and atPARP1-E960K and atPARP1-E960Q mutants. **(B)** Analysis of the PAR-DNA products generated by atPARP2-WT and atPARP2-E614K mutant. Arrows indicate HMW and LMW PAR–DNA products and free oligonucleotides. For more details, see section “Materials and Methods.”

To ensure that the observed DNA repair activities of recombinant atPARPs are not due to trace contamination by either bacterial host proteins or other unknown factors, we have constructed site-directed mutants of atPARP1 and atPARP2 and then purified them using the same scheme as for the wild-type proteins. The highly conserved catalytic E960 in atPARP1 and E614 in atPARP2 were replaced by either lysine (K) or glutamine (Q) resulting in single substitution mutants: atPARP1^E960K^, atPARP1^E960Q^, and atPARP2^E614K^. It should be noted that in the human PARP1 protein, the corresponding mutations E988Q and E988K strongly reduce > 40-fold the enzyme activity and convert PARP1 into a mono-ADP-ribosyl-transferase (Marsischky et al., 1995; Rolli et al., 1997). The purified atPARP1^E960K^, atPARP1^E960Q^, and atPARP2^E614K^ mutant proteins were incubated with the 5′-^32^P-labeled Exo15•Rex12T^Rec^ (S7) and p10•RT-A^Nick^ (S18) duplexes, respectively, to measure the DNA ADP-ribosylation activity. The results revealed that the atPARP1^E960K^ mutant protein completely lost DNA ADP-ribosylation activities (Figure 5A, lanes 8–11), whereas, as expected, the atPARP1^E960Q^ mutant exhibited robust DNA MARylation activity (lanes 12–14). Noteworthy, at higher protein concentration, the atPARP1^E960Q^ mutant was able to synthesize short ADP-ribose oligomers, but rather in a distributive manner (lane 15). In control reactions, human PARP1 and WT atPARP1 synthesized mainly HMW PAR–DNA products (Figure 5A, lanes 1 and 6–7, respectively). The atPARP2^E614K^ mutant at low protein concentration did not show detectable DNA PARylation activity (Figure 5B, lanes 7–8), but exhibited a very weak DNA mono-ADP-ribosylation (MARylation) activity at higher protein concentration (lanes 9–10). In control reactions, human PARP2 and WT atPARP2 showed efficient DNA PARylation activity (Figure 5B, lane 2 and lanes 3–6, respectively). Altogether, these results indicate that the highly conserved E960 of atPARP1 and E614 of atPARP2 are essential for DNA PARylation activities of plant enzymes and that the preparations of plant proteins used in this study are not contaminated by some unknown bacterial ADP-ribose transferases.

### Analysis of the Structure and Composition of PAR–DNA Adducts

**FIGURE 6:**
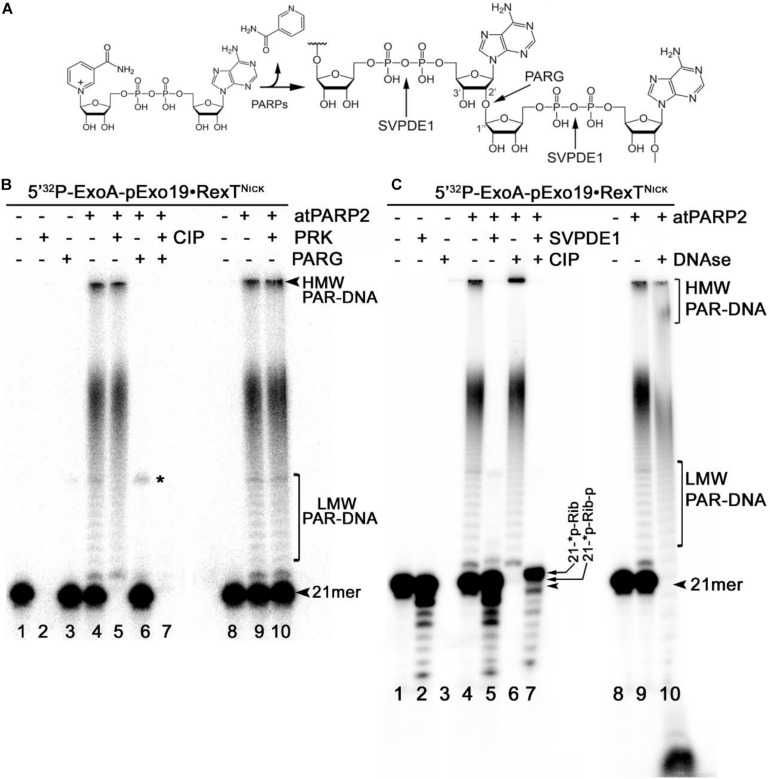
Analysis of the products of enzymatic digestion of the PAR–DNA adducts. PAR–DNA products were generated by incubation of 20 nM 5′-[^32^P]-ExoA pExo19•RexT^Nick^ oligonucleotide duplex (S13) with 250 nM atPARP2 in the presence of 1 mM NAD^+^ for 30 min at 37°C. After reactions, the samples were heated for 20 min at 80°C and then incubated in the presence of either 50 pg•μl^–1^ PARG (in ADPR buffer), or 50 μg•ml^–1^ proteinase K, or 0.1 U SVPDE1 (in SVPDE1 buffer) or 10 U CIP (in CIP buffer) for 60 or 30 min at 37°C, respectively. **(A)** Graphical representation of the formation of poly(ADP-ribose) polymer and enzyme cleavage sites. **(B)** Denaturing PAGE analysis of the products of PARG- and proteinase-catalyzed digestion of the 5′-[^32^P]-labeled PAR–DNA products. **(C)** Denaturing PAGE analysis of the products of SVPDE-, CIP-, and DNase-catalyzed digestion of the 5′-[^32^P]-labeled PAR–DNA products. Arrows indicate HMW and LMW PAR–DNA products and the 21-mer free oligonucleotide. Asterisk indicates a non-specific ligation product produced by *E. coli* NAD^+^-dependent DNA ligase A.

The mammalian PARPs use DNA strand break termini containing either the terminal phosphate residues or the 3′-terminal cordycepin moiety with 2′ hydroxyl group as acceptor residues for ADP-ribose chain synthesis. To verify whether plant PARPs employ the same mechanisms to modify DNA, we incubated 5′-[^32^P]-labeled DNA duplexes with atPARPs and then treated the reaction products with various enzymes including (i) PAR glycohydrolase (PARG) that hydrolyzes ribose–ribose *O*-glycosidic (1″→2′) bonds in PAR polymers to produce monomeric ADP-ribose (Figure 6A); (ii) CIP that removes phosphate groups from the 5′ and 3′ ends of DNA strand breaks; (iii) snake venom phosphodiesterase 1 (SVPDE1), which digests DNA in the 3′→5′ direction producing dNMPs and cleaves pyrophosphate bonds in a PAR polymer to generate the 2′-(5″-phosphoribosyl)-5′-adenosine monophosphate (pRib-AMP) compound as an end product (Figure 6A); (iv) deoxyribonuclease I from bovine pancreas (DNAse I) that non-specifically cleaves ssDNA and dsDNA to produce di, tri, and oligomer DNA fragments; and (v) proteinase K, a highly efficient non-specific serine protease that can efficiently digest majority of proteins.

Incubation of the 5′-[^32^P]-labeled ExoA•RexT^Nick^ (S13) duplex with atPARP2 resulted in the formation of LMW and HMW PAR–DNA complexes (Figure 6B, lanes 4 and 9). As expected, the treatment of PAR–DNA complexes with PARG resulted in complete disappearance of the ADP-ribosylated DNA and restoration of the original 21-mer fragment (lane 6), indicating that PARG can completely remove ADP-ribose moieties attached to DNA. However, these PAR–DNA complexes were resistant to proteinase K, SDS, and heat treatment (lane 10), suggesting that the PAR polymer is not attached to the atPARP2 protein. Indeed, the DNAse I treatment of PAR–DNA complexes resulted in a faster mobility of LMW and HMW products (Figure 6C, lane 10), indicating that PAR polymers are linked to the labeled 21-mer fragment. Noteworthy, CIP dephosphorylates free 5′-[^32^P]-labeled ExoA•RexT^Nick^ duplex (Figure 6B, lane 2 and Figure 6C, lane 3), but not the [^32^P]-labeled LMW and HMW PAR–DNA adducts generated by atPARP2 (Figure 6B, lane 5 and Figure 6C, lane 6), indicating that the 5′-terminal P of PARylated oligonucleotides is not accessible to the phosphatase. Moreover, the efficient shielding of 5′-[^32^P] groups from CIP, by the short ADP-ribose oligomers attached to ExoA oligonucleotide in LMW PAR–DNA products (Figure 6B, lane 5 and Figure 6C, lane 6), suggests that these DNA 5′-phosphates are protected *via* covalent phosphodiester bond between 5′P and C1′ of ADP-ribose.

Under the reaction conditions used, SVPDE1 degrades the free 5′-[^32^P]-labeled ExoA•RexT^Nick^ duplex in the 3′→5′ direction, resulting in the appearance of a fast migrating ladder with bands below the 21-mer fragment (Figure 6C, lane 2). Incubation of the PARylated ExoA•RexT^Nick^ duplex with SVPDE1 resulted in a disappearance of LMW and HMW PAR–DNA complexes (lane 5), indicating that the enzyme degrades PAR by cleaving the pyrophosphate bonds within a polymer chain. The SVPDE1-catalyzed hydrolysis of [^32^P]-labeled PAR–DNA products converted the LMW and HMW complexes back to a free DNA fragment, which migrates somewhat similar to a free 21-mer (lane 5). We propose that this SVPDE1-generated 21-mer fragment still contains the phosphoribosyl moiety left after the hydrolysis of the last ADP-ribose monomer linked to the terminal DNA phosphate residue at the 5′ end of ExoA. In agreement with this, the combined treatment of 5′-[^32^P]-labeled PAR–DNA products with SVPDE1 and CIP resulted in the appearance of a band (lane 7) that migrated more slowly than free 21-mer 5′-[^32^P]-labeled ExoA (lane 1). This result strongly suggests the presence of a protecting ribose moiety at the 5′ end of the ADP-ribosylated ExoA that remains after the removal of PAR and phosphate residue by SVPDE1 and CIP, respectively.

Next, we examined the structure and composition of PAR–DNA adducts generated by atPARP1. For this, the 5′-[^32^P]-labeled PARylated Exo15•Rex12T^Rec^ (S7) oligonucleotide duplexes were incubated with PARG, CIP, DNAse I, and PRK. As expected, PARG treatment of PARylated DNA, but not that of PRK, completely restored the native structure of the 15-mer oligonucleotide (Supplementary Figure S3). PARylated Exo15•Rex12T^Rec^ duplexes, contrary to free oligonucleotides, were resistant to CIP and DNAse I treatments (Supplementary Figure S3). These results indicate that both atPARPs ADP-ribosylate DNA oligonucleotides in a similar manner by generating structurally similar PAR–DNA adducts. Thus, we can conclude that plant PARPs, similar to their mammalian counterparts, catalyze covalent attachment of an ADP-ribose unit to DNA termini *via* a phosphodiester bond between DNA terminal phosphate residue and C1′ of ADP-ribose.

### Nucleoside Diphosphate-Linked Moiety X Hydrolase Cleaves the PAR–DNA Complexes to Generate the Phosphoribosylated DNA Adducts

Previously, it was demonstrated that nucleoside diphosphate-linked moiety X (Nudix) hydrolases can act on a free ADP-ribose residue (and on a PAR polymer attached to a protein) by hydrolyzing the pyrophosphate bonds (Mildvan et al., 2005). In addition, Nudix hydrolases can cleave a long PAR polymer attached to DNA (Palazzo et al., 2015; Talhaoui et al., 2016; Belousova et al., 2018); here, this property was exploited to further characterize DNA ADP-ribosylation catalyzed by plant PARPs. For this, the 5′-[^32^P]-labeled ExoA•RexT^Nick^ (S13) and Exo15•Rex12T^Rec^ (S7) duplexes were ADP-ribosylated by atPARP1 and atPARP2, respectively, and the resulting PAR–DNA complexes were incubated with an excess amount of the human Nudix hydrolase, NUDT16, and the products of reaction were analyzed by denaturing PAGE.

**FIGURE 7:**
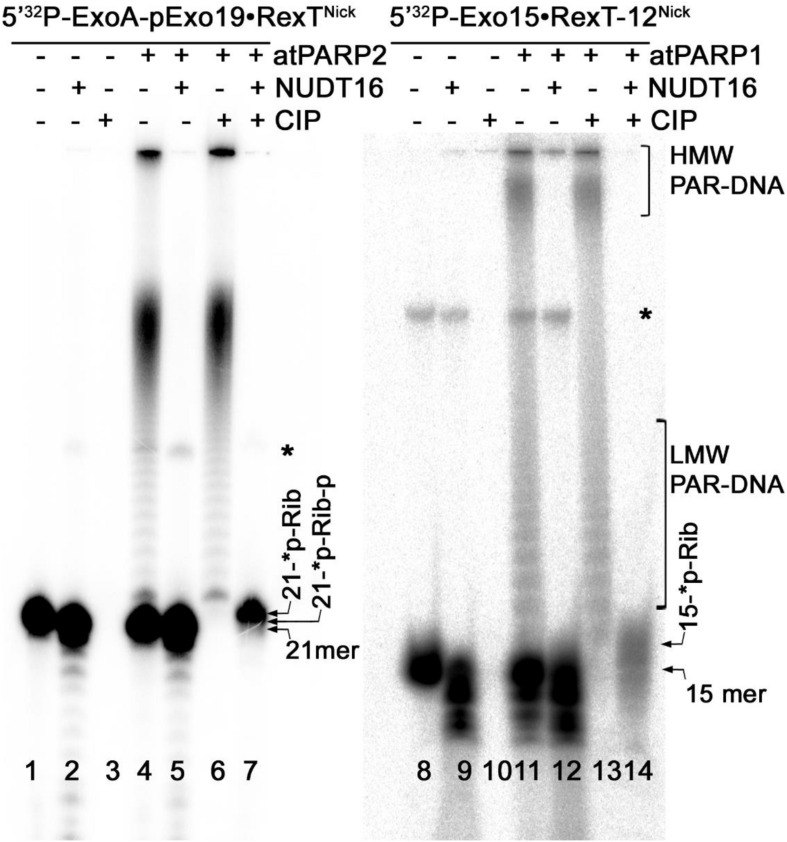
Denaturing PAGE analysis of the products of NUDT16- and CIP-catalyzed hydrolysis of the PAR–DNA adducts generated by atPARP1 and atPARP2. The 20-nM 5′-[^32^P]-labeled ExoA pExo19•RexT^Nick^ duplex (S13) was incubated with 250 nM atPARP2 and 1 mM NAD^+^, and the 20-nM 5′-[^32^P]-labeled Exo15•Rex12T^Rec^ duplex (S7) was incubated with 250 nM atPARP1 and 1 mM NAD^+^ at 37°C for 30 min. After incubation with atPARPs, the samples were heated for 20 min at 80°C and the resulting [^32^P]-labeled HMW products were further incubated with 20 μM NUDIX16. Arrows indicate phosphoribosylated (Rib-p), ribosylated (Rib), and native [^32^P]-labeled 21-mer and 25-mer oligonucleotides, “*p” stands for a labeled phosphate residue. Asterisk indicates a nonspecific ligation product produced by *E. coli* NAD^+^-dependent DNA ligase A. For more details, see section “Materials and Methods.”

Incubation of the 5′-[^32^P]-labeled free oligonucleotide duplexes with an excess amount of NUDT16 resulted only in slight degradation of the 21-mer oligonucleotide, whereas the shorter 15-mer oligonucleotide degraded more strongly (Figure 7, lanes 2 and 9, respectively), suggesting that the human Nudix hydrolase contains a weak non-specific nucleolytic cleavage activity. NUDT16 completely degraded the PAR–DNA adducts and generated distinct DNA fragments that migrated similar to free 21-mer and 15-mer oligonucleotides (lanes 5 and 12). The mechanism of action of NUDT16 on PAR suggests that a phosphoribosyl (pRib) moiety attached to the 5′-terminal [^32^P] residue at DNA termini (21-^∗^p-Rib-p and 15-^∗^p-Rib-p, where the asterisk denotes a radioactive ^32^P residue) should remain after NUDT16-catalyzed hydrolysis of pyrophosphate bonds of the ADP-ribose unit was covalently linked to DNA. As expected, the treatment of NUDT16-derived DNA oligonucleotides with CIP resulted in the appearance of the distinct 5′-monoribosylated 21- and 15-mer DNA fragments (lanes 7 and 14, respectively), which migrated more slowly than free 21- and 15-mer DNA oligonucleotides (lanes 1 and 8, respectively) and the 5′-mono-phosphoribosylated 21- and 15-mer NUTD16 products (lanes 5 and 12, respectively). These results suggest that NUTD16 generated the 21-^∗^p-Rib-p and 15-^∗^p-Rib-p fragments by hydrolysis of the PARylated 21- and 15-mer oligonucleotides, respectively. After that, CIP dephosphorylated NUTD16 products to generate monoribosylated 21-^∗^p-Rib and 15-^∗^p-Rib fragments which still contain ^32^P residue. Noteworthy, CIP did not remove the 5′-[^32^P] residue in PARylated DNA fragments, even after hydrolysis of the PAR polymer by NUDT16 (lanes 5–12), indicating that the remaining ribose sugar moiety protects 5′P in the 5′-[^32^P]-ExoA(Exo15)-p^∗^-Rib oligonucleotide. These results further confirms that the plant PARPs catalyze covalent attachment of an ADP-ribose unit to DNA *via* a phosphodiester bond between DNA 5′P and C1′ of ADP-ribose.

### Identification of the ADP-Ribose–DNA Adducts by Matrix-Assisted Laser Desorption Ionization Time-of-Flight Mass Spectrometry

In the above data, a putative molecular mechanism of *Arabidopsis* atPARP-catalyzed DNA PARylation is revealed from the migration pattern of end-labeled DNA fragments in a denaturing PAGE (Figures 6, 7). To further substantiate the mechanism of action of atPARP enzymes on duplex oligonucleotides, we characterized the nature of PAR–DNA adducts by MALDI-TOF MS analysis of the PARylated DNA products. For this purpose, we selected atPARP2 as the most efficient enzyme and constructed cold non-radioactive 30-mer nicked duplex oligonucleotide (referred to here as p10•RT-A^Nick^ or S18), composed of a 30-mer (RT-A) template strand and two 5′-phosphorylated complementary strands: 10-mer (p10) and 19-mer (p19), as DNA substrate (Supplementary Table S1). It should be noted that, when acting upon p10•RT-A^Nick^, atPARP2 generates mainly LMW PAR–DNA products, which migrate as DNA ladders in the denaturing gel, indicating the presence of short ADP-ribose oligomers (1–20 units) linked to the 10-mer fragment (Supplementary Figure S4). Furthermore, short, low-molecular-weight oligonucleotides (such as 10-mer in p10•RT-A^Nick^) have in general higher probability of detection by MALDI-TOF MS as compared with their long, high-molecular-weight oligonucleotide analogs (such as 21-mer in ExoA•RexT^Nick^, or S13) (Nordhoff et al., 1996); thus, the employment of p10•RT-A^Nick^ allowed us to significantly increase the sensitivity of mass spectrometry.

**FIGURE 8:**
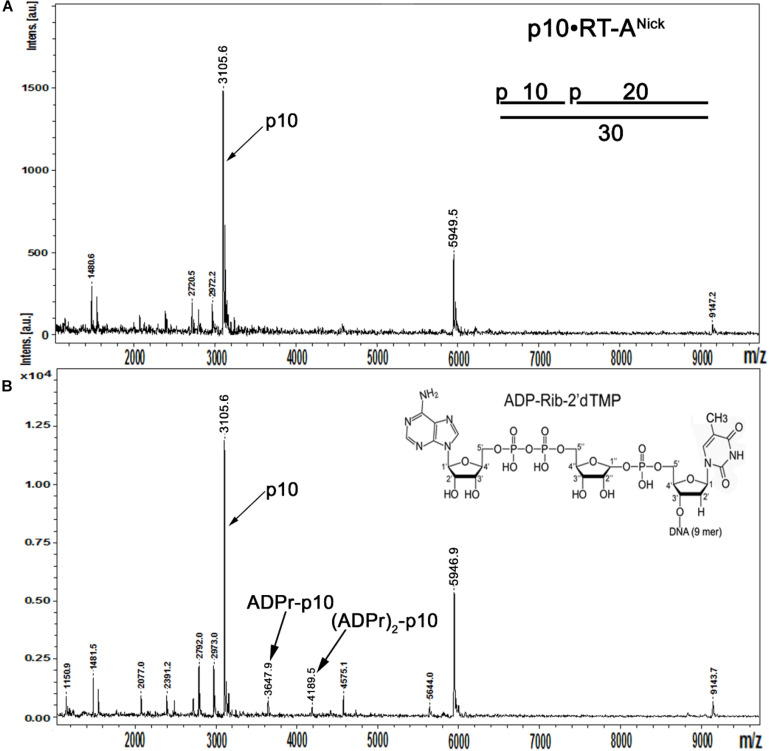
MALDI-TOF MS analysis of the mono- and poly-ADP-ribosylated oligonucleotides generated by atPARP2. Five micromolars of the cold 5′-phosphorylated non-radioactive p10•RT-A^Nick^ duplex (S18) was ADP-ribosylated in the presence of 2.5 μM of atPARP2 and 1 mM NAD^+^ at 37°C for 1 h. **(A)** MALDI-TOF spectrum of the control mock-treated cold 5′-phosphorylated RT^Nick^ duplex. **(B)** MALDI-TOF spectrum of the unpurified atPARP2 reaction products supplemented with the purified 10-mer p-10-RT-poly-ADP-ribose fragment as a size marker. For more details, see section “Materials and Methods.”

MALDI-TOF analysis of the mock-treated p10•RT-A^Nick^ duplex showed the presence of two major peaks at [M-H]^–^ = 3,105.6 Da and [M-H]^–^ = 5,949.5 Da corresponding to the phosphorylated 10-mer and 19-mer oligonucleotides, as well as a minor peak corresponding to 30-mer RT-A oligonucleotide (Figure 8A). Analysis of the mass spectra of the atPARP2 ADP-ribosylated p10•RT-A^Nick^ duplex oligonucleotide revealed two monocharged peaks at [M-H]^–^ = 3,647.9 and 4,189.5 Da corresponding to the 5′-phosphorylated 10-mers that contain one and two ADP-ribose residues, respectively (calculated mass, 3,647 and 4,187 Da) (Figure 8B). These results indicate that atPARP2 catalyzes covalent attachment of ADP-ribose residues to the 5′-phosphorylated 10-mer (p10) oligonucleotide. In conclusion, these data are in good agreement with those obtained through the analysis of the PAR–DNA products on denaturing PAGE (Figures 6, 7) and unambiguously confirm the formation of the covalent PAR–DNA adducts by plant PARPs.

### The Switch of atPARP2 Substrate Specificity Depends on the Presence of Terminal Phosphates and DNA Duplex Configuration

Depending on the specific configurations of multiple closely spaced DNA strand breaks, mammalian PARP1–3 can switch their substrate specificity from protein to DNA only ADP-ribosylation (Zarkovic et al., 2018; Matta et al., 2020). In order to assess the relative efficiency of atPARP2-catalyzed auto- (protein) versus DNA ADP-ribosylation activities, we used non-radioactive (cold), non-phosphorylated, nicked 40-mer ExoA•RexT^Nick^ duplex as a cofactor and cold phosphorylated pExoA•RexT^Nick^ duplex (S13) as a DNA substrate. It should be noted that the pExoA•RexT^Nick^ duplex containing 5′-phosphorylated 21-mer fragment is prone to covalent ADP-ribosylation by human and plant PARPs at the 5′-terminal phosphate residue, whereas the ExoA•RexT^Nick^ duplex containing non-phosphorylated 21-mer fragment is not a substrate for ADP-ribosylation by PARPs. Importantly, both DNA duplexes can activate auto-ADP-ribosylation of mammalian and plant PARPs. To avoid the formation of long PAR polymers, we incubated 10-fold the molar excess of DNA duplexes (10 μM) over atPARP2 (1 μM) in the presence of limited amount of radioactively labeled [adenylate-^32^P]NAD^+^ (1 μM). We expected that under this particular reaction conditions, atPARP2 and other PARPs would favor the MARylation, rather than PARylation, of proteins and DNA. Human PARP3 was used as a control, because when acting upon pExoA•RexT^Nick^ duplex, this enzyme switches its substrate specificity from auto- to only DNA MARylation (Zarkovic et al., 2018). However, if PARP3 acts upon the non-phosphorylated ExoA•RexT^Nick^ duplex as DNA cofactor, it switches to auto-MARylation.

**FIGURE 9:**
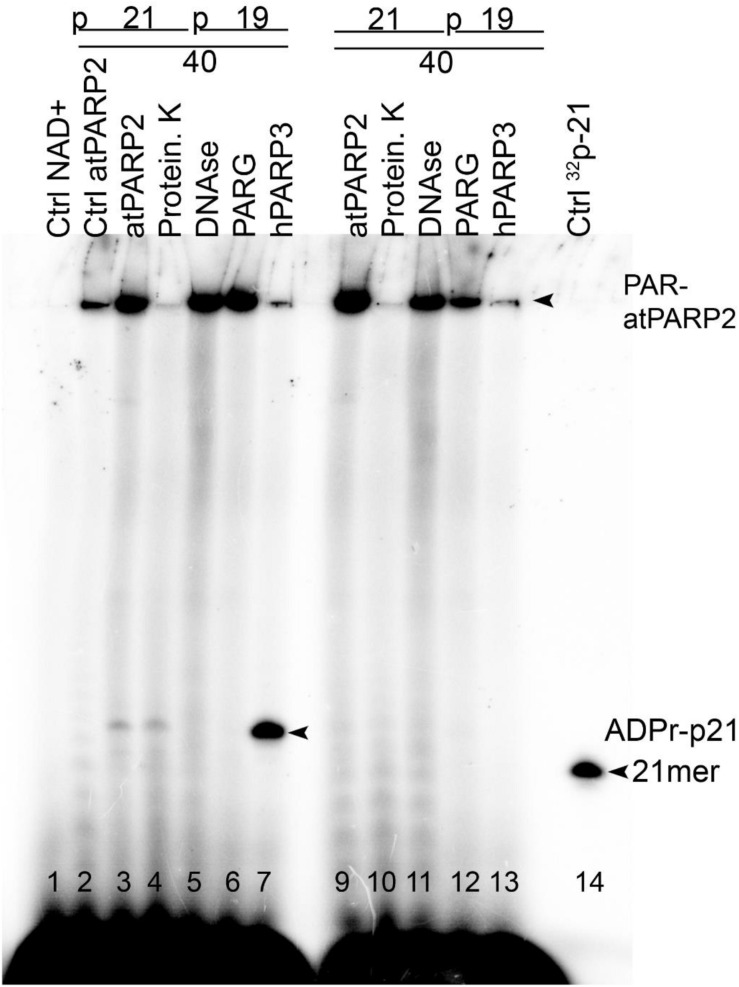
Comparison of the relative efficiency of atPARP2-catalyzed auto- and DNA ADP-ribosylation; 1 μM atPARP2 and 50 nM human PARP3 were incubated with 10 μM cold oligonucleotide duplexes in the presence of 1 μM [adenylate-^32^P]-NAD^+^ for 30 min at 37°C. The reaction products were analyzed by denaturing PAGE. Arrows indicate PAR–DNA, mono-ADPr-p21 mer products and free 21-mer oligonucleotide. For more details, see section “Materials and Methods.”

As shown in Figure 9, human PARP3 incubated with cold pExoA•RexT^Nick^ duplex (S13) and [adenylate-^32^P]NAD^+^ generated MARylated 21-mer pExoA fragment (lane 7), which migrated slower than the 21-mer size marker (lane 13), whereas no DNA MARylation occurred when PARP3 was incubated with the non-phosphorylated 40-mer nicked duplex (lane 12). On the other hand, incubation of atPARP2 with cold phosphorylated pExoA•RexT^Nick^ and radioactive NAD^+^ resulted in the generation of a major band at the top of the gel, smears, and a minor band migrating similar to MARylated 21-mer pExoA fragment (lane 3). Formation of the major band at the top of the gel suggests auto-ADP-ribosylation of atPARP2, whereas the appearance of the smearing and modified 21-mer pExoA fragment suggests PARylation and MARylation of DNA, respectively. In agreement with these, treatment of the atPARP2 reaction products by proteinase K resulted in the disappearance of a major band and a dramatic decrease in smearing, but not the minor band (lane 4). As expected, DNAse I and PARG treatments resulted in the complete disappearance of the minor MARylated 21-mer pExoA fragment, but not the major band (lanes 5 and 6). These results suggest that atPARP2 when acting upon phosphorylated nicked duplex can partially switch its substrate specificity from protein to DNA, but keeps its preference for auto-ADP-ribosylation.

Incubation of atPARP2 with cold non-phosphorylated ExoA•RexT^Nick^ duplex and radioactive NAD^+^ resulted in the generation of a major band at the top of the gel and some smearing, and no discrete bands migrating between 21-mer pExoA fragment and the top of the gel were observed (Figure 9, lane 8). Proteinase K treatment, but not that of DNAse I and PARG, resulted in the complete loss of a major band, suggesting auto-ADP-ribosylation of atPARP2 (lane 9 versus lanes 10–11). Noteworthy, the PARG treatment resulted in a significant decrease of the top band and smearing, suggesting the presence of PARylated atPARP2 protein and free PAR polymer (lane 11). Taken together, these results strongly suggest that DNA fragments containing multiple closely spaced phosphorylated strand break termini are prone to covalent modifications by plant PARP proteins.

**FIGURE 10:**
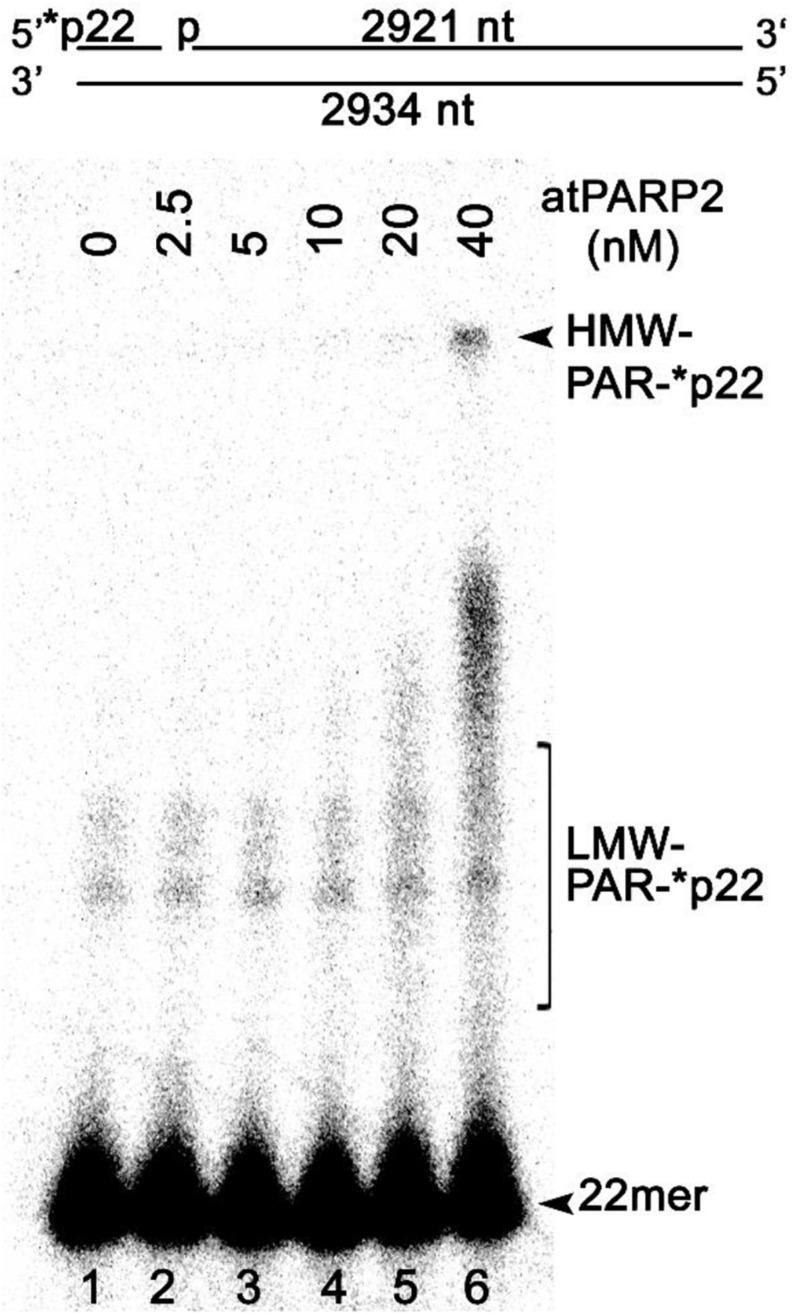
AtPARP2 poly-ADP-ribosylates linear plasmid 2.3 kb DNA fragment; 1 nM 5′-[^32^P]-labeled linearized nicked pML2 plasmid DNA was incubated with 2.5–40 nM atPARP2 under the standard reaction conditions. After incubation, the reaction products analyzed by denaturing PAGE. Arrows indicate HMW and LMW PAR–DNA products and free oligonucleotides. For more details, see section “Materials and Methods.”

Previously, it has been demonstrated that mammalian PARPs can ADP-ribosylate with high efficiency long plasmid DNA fragments containing an SSB in close proximity to DSB termini (Zarkovic et al., 2018). To examine whether atPARP2 could ADP-ribosylate high-molecular-weight DNA fragments, we constructed a linear 2,934-bp plasmid-based DNA fragment containing a single nick 22 nt away from the 5′-[^32^P]-labeled blunt-ended DSB (Figure 10). The 40-nM atPARP2 protein exhibited robust ADP-ribosylation of the 5′-phosphorylated 22-mer fragment which positioned between nick and DSB end (lane 6), suggesting that the DNA ADP-ribosylation activity of *Arabidopsis* PARPs is not limited to short oligonucleotide duplexes, but extends to high-molecular-weight DNA structures. Taken together, these data showed that plant PARPs have broad DNA substrate specificities similar to that of mammalian counterparts, although atPARP2 has more efficient DNA ADP-ribosylation activity as compared with that of atPARP1.

### Search for PAR–DNA Adducts in Plant Genomic DNA After a Genotoxic Treatment

**FIGURE 11:**
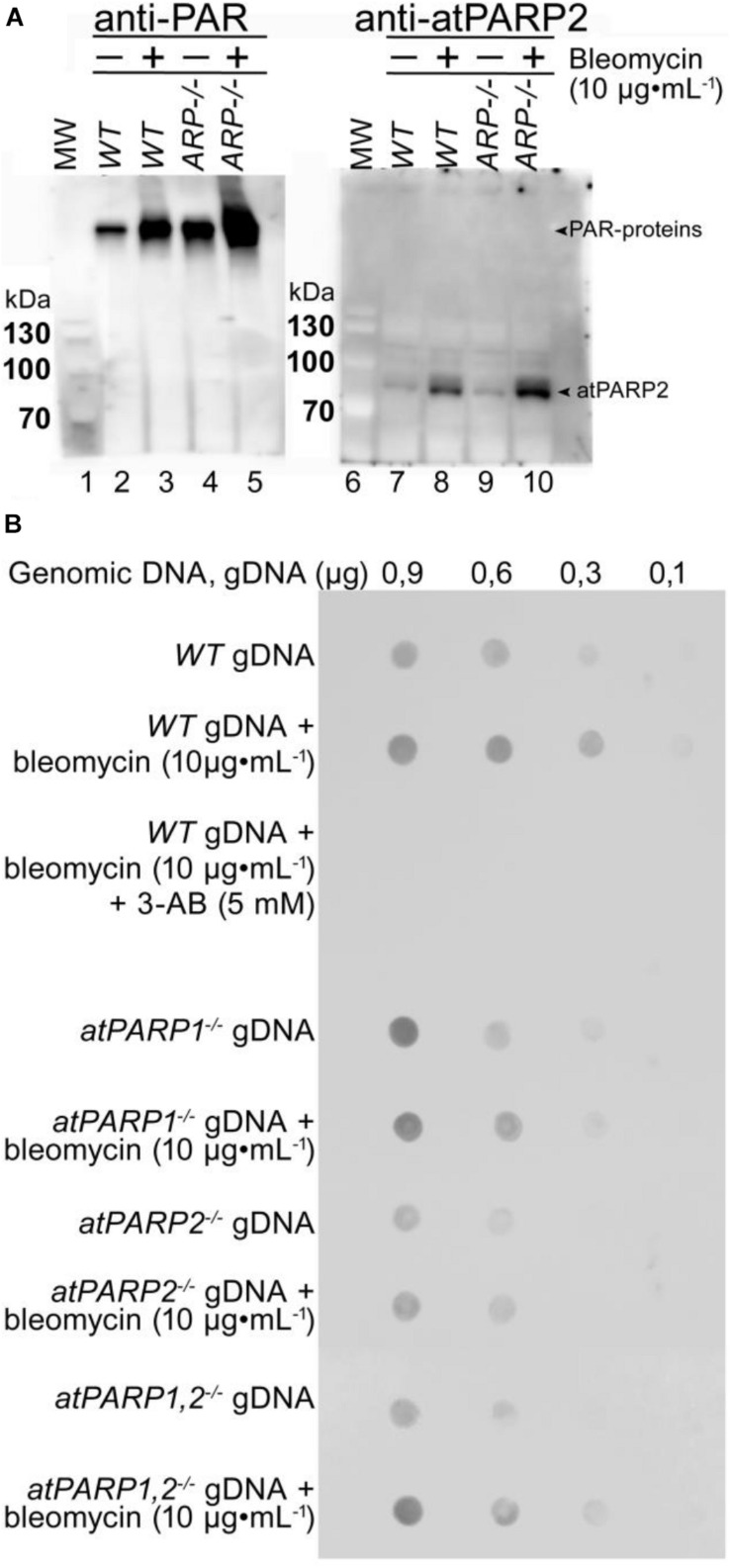
Detection of ADP-ribosylation in plant cells. **(A)** Western blot analysis of protein ADP-ribosylation in WT and mutant plant extracts. Wild-type Col-0 and AP endonuclease-deficient *ARP*^–/–^ mutant seeds were grown on MS agar medium. Two-week-old *Arabidopsis* plants were transferred to 10 μg•ml^–1^ of bleomycin for 18 h. Total proteins were extracted from whole plants, separated by SDS-PAGE, and analyzed by immunoblotting using anti-PAR and anti-atPARP2 antibodies. Arrows indicate PARylated proteins and atPARP2 protein. **(B)** Detection of PAR–DNA adducts in gDNA extracted from 14-day-old seedlings grown under either normal conditions or genotoxic stress. Different quantities of gDNA in TE buffer (10 mM Tris-Cl pH 8.0, 1 mM EDTA) were spotted onto a nylon membrane, followed by mouse monoclonal anti-poly(ADP-ribose) antibody 10H dot blotting. For more details, see section “Materials and Methods.”

To measure ADP-ribosylation *in vivo*, cell-free extracts and cellular DNA from *Arabidopsis* were analyzed by immunoblotting using the homemade polyclonal rabbit antibodies against atPARP2 and commercial anti-PAR monoclonal antibody. The *Arabidopsis* PARPs were activated by plant exposure to bleomycin (10 μg•ml^–1^). To measure protein ADP-ribosylation, soluble cell-free extracts form *A. thaliana WT* and *arp*^–/–^ (AP endonuclease-deficient) mutant were separated on SDS-PAGE gel, and then Western blotted using anti-PAR and anti-atPARP2 antibodies (Figure 11A). Anti-PAR antibody detected a weak PARylation activity in non-treated WT plants, which strongly increased after exposure of the plants to bleomycin (lanes 1 and 2, respectively), suggesting that DNA strand breaks induced by bleomycin activate PARP-catalyzed ADP-ribosylation. Noteworthy, the level of PARylation in non-treated *arp*^–/–^ mutant plants (lane 3) was significantly higher as compared with *WT* (lane 1), suggesting the accumulation of unrepaired DNA strand breaks in the absence of major plant AP endonuclease and activation of the DNA damage signaling pathway. As expected, the exposure to bleomycin of the *arp*^–/–^ mutant leads to a significantly higher level of PARylation (lane 4) as compared with both control non-treated *arp*^–/–^ plants (lane 3) and even treated *WT* plants (lane 2), suggesting that ARP participates in the repair of bleomycin-induced DNA strand breaks. Western blot using anti-atPARP2 antibodies showed bleomycin-induced overexpression of the atPARP2 protein in *WT* and *arp*^–/–^ mutant plants (lanes 8 and 10) as compared with non-treated controls (lanes 7 and 9). Again, the overexpression of atPARP2 in response to DNA damage was significantly higher in *arp*^–/–^ mutant plants as compared with *WT* ones (lane 10 versus 8). Overall, these results suggest that in the response to DNA damage, plants activate protein ADP-ribosylation and overexpress the atPARP2 protein.

To examine a putative DNA ADP-ribosylation activity in living cells, gDNA were isolated from *WT* and *atPARP*-deficient plants treated or not with bleomycin and examined for the presence of PAR. The gDNA were repeatedly purified, including extensive RNAse A and proteinase K treatments followed by phenol/chloroform extraction, and then analyzed by the dot blotting technique using the mouse anti-PAR monoclonal antibody and the rabbit monoclonal anti-pan-ADP-ribose binding reagent (MABE1016). Immunodot blot analysis of 0.9 and 0.6 μg of the gDNA isolated from control non-treated *WT*, *atPARP1^–/–^*, *atPARP2^–/–^*, and double mutant *atPARP1^–/–^ atPARP2^–/–^* revealed the presence of PAR in all samples (Figure 11B and Supplementary Figure S5). The gDNA purified from bleomycin-treated plants showed increased presence of PAR, as compared with non-treated controls. Nevertheless, the presence of gDNA-associated PAR in control non-treated *WT* and *atPARPs^–/–^* mutant plant suggest two possibilities: (i) contamination of the purified gDNA with PARylated peptides that are tightly bound or cross-linked to DNA and highly resistant to proteinase K treatments or with free ADP-ribose oligomers which may exist in non-covalent intertwined complexes with gDNA and (ii) cross-reactivity or non-specific recognition of some DNA structures present in plant gDNA by monoclonal anti-PAR antibodies. Taken together, these results demonstrate that the PAR-specific antibodies, although good to detect PARylated proteins, have very limited use to detect covalent PAR–DNA adducts because they are not able to specifically recognize DNA nucleotide linked to ADP-ribose. Thus, new types of antibodies are required to detect ADP-ribosylated DNA in living cells that can recognize both ADP-ribose and DNA nucleotide with high specificity.

## Discussion

In the present work, by using *in vitro* approaches, we demonstrated that plant *A. thaliana* poly(ADP-ribose) polymerases atPARP1 and atPARP2, similar to their mammalian counterparts, ADP-ribosylate DNA strand break termini harboring terminal phosphate residues. Particularly, atPARP1, like human PARP1, preferentially PARylates recessed DNA duplex and exhibits the following order of preference: Rec > Nick > Gap duplexes. On the other hand, atPARP2, like mammalian PARP2, PARylates Nick and Gap duplexes more efficiently than recessed duplex and displays the following order of preference: Nick > Gap > Rec duplexes (Figure 2). Kinetics of DNA PARylation and optimal concentrations of NAD^+^ and enzymes were determined (Figure 3 and Supplementary Figure S2). We further substantiated the DNA substrate requirements for the efficient ADP-ribosylation of DNA strand breaks by plant PARPs (Figure 4). Noteworthy, contrary to mammalian enzymes, atPARP2 exhibited higher DNA PARylation activity, than atPARP1, on the majority of DNA substrates tested. Nevertheless, the atPARP1, but not atPARP2, was able to PARylate recessed DNA duplex containing short 15-mer oligonucleotide with 5′-terminal phosphate, suggesting that these plant enzymes have non-overlapping DNA substrate specificities. It should be stressed that the plant PARPs were particularly sensitive to the distance that separate DSB and SSB (presented in the form of nick, gap, or ssDNA tail) in a DNA duplex. For example, atPARP1 exhibited preference for DNA substrates containing two strand breaks separated by 1.5 turns of helix, whereas atPARP2 preferred the distance of 1 or 2 turns of helix (Figure 4). Thus, the presence of multiple closely spaced DNA strand breaks, their comparative positioning, and the nature of 5′ and 3′ termini in the DNA substrate are essential for the atPARP-catalyzed DNA ADP-ribosylation. Overall, except higher activity of atPARP2, the substrate specificities of plant atPARP1 and atPARP2 proteins were very similar to that of their mammalian counterparts PARP1 and PARP2, respectively.

The plant atPARPs share structural similarity with other PARP family members and contain a highly conserved catalytic triad “H-Y-E” in their ART domains. In this study, single substitution mutants—atPARP1^E960K^, atPARP1^E960Q^, and atPARP2^E614K^—in which a highly conserved glutamic acid residue in the catalytic triad was replaced by lysine or glutamine, were characterized for DNA ADP-ribosylation activity. As expected, all plant mutant atPARPs, similar to the corresponding mammalian mutants, have greatly reduced DNA PARylation activities (Figure 5). Nevertheless, atPARP1^E960Q^ and atPARP2^E614K^ mutants exhibited from robust to very weak DNA MARylation activity, respectively. Thus, these results demonstrate that highly conserved glutamic acid residue in the catalytic triad of plant atPARPs is required for DNA PARylation activities and that the preparations of recombinant PARP proteins are not contaminated by some uncharacterized host ADP-ribose transferases.

Biochemical analysis of the structure and composition of PAR–DNA adducts generated by plant PARPs, using the following enzymes: PARG, CIP, SVPDE1, DNAse I and PRK, revealed that similar to their mammalian counterparts, atPARPs utilize the 5′-terminal DNA phosphates as acceptor residue to covalently attach the ADP-ribose unit to synthesize the PAR polymer (Figure 6 and Supplementary Figure S3). We further substantiated the molecular mechanism of the plant atPARP-catalyzed DNA ADP-ribosylation by identifying the products of PAR–DNA degradation with human Nudix hydrolase, NUDT16. NUDT16 cleaves the PAR polymer attached to [^32^P]-labeled oligonucleotide duplex and generates ribosylated DNA fragment, in which the terminal phosphate residue is protected from dephosphorylation by CIP (Figure 7). These results indicate that atPARPs transfer ADP-ribose unit to terminal DNA phosphate residue at the strand break termini to generate a phosphodiester bond between DNA 5′P and C1′ of ADP-ribose. The putative molecular structure of ADP-ribose–p-DNA adduct was further confirmed by MALDI-TOF MS analysis of the ADP-ribosylated DNA fragments (Figure 8 and Supplementary Figure S4). The mass spectra of the atPARP2 ADP-ribosylated p10•RT-A^Nick^ duplex oligonucleotide (S18) showed the presence of two new peaks corresponding to the 5′-phosphorylated 10-mers containing one and two ADP-ribose residues (Figure 8B). Thus, the mass spectrometry data and biochemical analysis demonstrate that the structure of the covalent PAR–DNA adducts generated by plant atPARPs is the same as that synthesized by mammalian PARP enzymes.

Depending on the structure of DNA, mammalian PARPs can switch their mode of action from auto-ADP-ribosylation to DNA ADP-ribosylation (Zarkovic et al., 2018; Matta et al., 2020). Here, we demonstrate that plant atPARP2 can acquire additional substrate specificity when acting on 5′-phosphorylated nicked DNA duplex (Figure 9). Contrary to human PARP3, atPARP2 did not completely switch from auto- to DNA ADP-ribosylation when acting on its preferred DNA substrate, but continue to act on both substrates: protein and DNA, with the preference for the former one. Under the experimental conditions used, the non-phosphorylated nicked DNA duplex activates atPARP2-catalyzed auto-ADP-ribosylation to a similar extent as the phosphorylated one. It is possible that a certain configuration of the phosphorylated strand break termini in a DNA substrate, not examined in this work, would enable more pronounced switch from auto- to DNA ADP-ribosylation in plant atPARP2. Importantly, similar to mammalian PARPs, the DNA ADP-ribosylation activity of plant atPARP2 is not limited to short duplex oligonucleotides, but is also efficient toward strand breaks within high-molecular-weight linear plasmid DNA (Figure 10), suggesting that in plants, covalent modification of DNA may occur in chromosomal context.

We attempt to examine a possible biological role of atPARP-dependent DNA ADP-ribosylation by immunoblotting of the purified genomic DNA from plants to detect PAR–DNA adducts. The results revealed that two commercial monoclonal anti-PAR antibodies recognize gDNA isolated from both control non-treated and bleomycin-treated plants and also wild-type and PARP-deficient plants (Figure 11 and Supplementary Figure S5), suggesting that the approach used in the present study lacks sufficient specificity to detect PAR–DNA adducts in gDNA. New more advanced tools are required to reliably distinguish the ADP-ribosylated DNA products from the ADP-ribosylated proteins and free PAR polymers in living cells.

Overall, the plant atPARP1 and atPARP2 contain less efficient DNA ADP-ribosylation activity as compared with their mammalian homologs PARP1 and PARP2. In addition, contrary to mammalian PARPs, atPARP2 is a major poly(ADP-ribose) polymerase in *Arabidopsis* and has higher activity than atPARP1. AtPARP-catalyzed DNA ADP-ribosylation strongly depends on the presence of closely spaced multiple DNA strand breaks, which are located within either 1.5 or 1.0 and 2.0 turns of helix. In summary, the finding that plant poly(ADP-ribose) polymerases can covalently modify the termini of DNA strand breaks by covalent attachment of PAR chains *in vitro* suggests that this property is universally conserved among eukaryotic PARPs and that in plants cellular DNA may undergo postreplicative modification in response to DNA damage.

## Data Availability Statement

The original contributions presented in the study are included in the article/Supplementary Material, further inquiries can be directed to the corresponding authors.

## Author Contributions

ST, AK, RG, YB, and AAI performed all the biochemical experiments. ST and AK performed all the plant experiments. AAI, MS, and AKB designed all the experiments. CS-P and DG performed the mass spectrometry analysis. ST, AAI, MS, and AKB wrote the manuscript.

## Conflict of Interest

The authors declare that the research was conducted in the absence of any commercial or financial relationships that could be construed as a potential conflict of interest.

## Footnotes

1 http://www.arabidopsis.org
