# Supplementary material for: The Arabidopsis thaliana Poly(ADP-Ribose) Polymerases 1 and 2 Modify DNA by ADP-Ribosylating Terminal Phosphate Residues
Source: Front Cell Dev Biol. 2020 Nov 26;8:606596. doi: 10.3389/fcell.2020.606596 (PMC7726343; doi:10.3389/fcell.2020.606596)
Supplement: Supplementary file 1 [file Data_Sheet_1.pdf]

SUPPLEMENTARY MATERIAL

The *Arabidopsis thaliana* poly(ADP-ribose) polymerases 1 and 2 modify DNA by ADP-ribosylating terminal phosphate residues

Sabira TAIPAKOVA[1], Aigerim KUANBAY[1,2], Christine Saint-Pierre[3], Didier Gasparutto[3], Yeldar BAIKEN[4,5], Regina GROISMAN[2], Alexander A. ISHCHENKO[2,*], Murat SAPARBAEV[1,2,*] and Amangeldy K. BISSENBAEV[1,*]

[1] Department of Molecular Biology and Genetics, Faculty of Biology and Biotechnology , al-Farabi Kazakh National University, 0530040, Almaty, Kazakhstan.

[2] Groupe «Mechanisms of DNA Repair and Carcinogenesis», Equipe Labellisée LIGUE 2016, CNRS UMR9019, Université Paris-Saclay, Gustave Roussy Cancer Campus, F-94805 Villejuif Cedex, France.

[3] Université Grenoble Alpes, CEA, CNRS, IRIG / SyMMES-UMR5819 / CREAB, F-38000 Grenoble, France.

[4] National Laboratory Astana, Nazarbayev University, Nur-Sultan, Kabanbay Batyr ave 53, 010000, Kazakhstan.

[5] School of Engineering and Digital Sciences, Nazarbayev University, Nur-Sultan, Kabanbay Batyr ave 53, 010000, Kazakhstan.

Supplementary Material Table S1. Sequences of the oligonucleotides and their duplexes used in this study[a].

Name	Oligonucleotides sequences and structures
S1	32P-<br>CACCGCGCCTCTGAATCTCTTTAAACCGCGCCCCTTAAGG 5'<br>RexT
S2	32P-<br>CACCGCGCCTCTGAATCTCTTTAAACCGTGCCCCTTAAGG 5'<br>Rex12T
S3	P- ExoA<br>5'GTGGCGCGGAGACTTAGAGAA<br>CACCGCGCCTCTGAATCTCTTTAAACCGCGCCCCTTAAGG 5'<br>RexT -32P
S4	P- Exo20<br>5'GTGGCGCGGAGACTTAGAGA-dAM 32P<br>CACCGCGCCTCTGAATCTCTTTAAACCGCGCCCCTTAAGG 5'<br>RexT
S5	32P- ExoA<br>5'GTGGCGCGGAGACTTAGAGAA<br>CACCGCGCCTCTGAATCTCTTTAAACCGCGCCCCTTAAGG 5'<br>RexT
S6	32P- ExoA P-<br>5'GTGGCGCGGAGACTTAGAGAA<br>CACCGCGCCTCTGAATCTCTTTAAACCGCGCCCCTTAAGG 5'<br>RexT
S7	32P- Exo15<br>5'GTGGCGCGGAGACTT<br>CACCGCGCCTCTGAATCTCTTTAAACCGTGCCCCTTAAGG 5'<br>Rex12T
S8	32P- ExoA P- Exo14<br>5'GTGGCGCGGAGACTTAGAGAA GCGCGGGGAATTCC<br>CACCGCGCCTCTGAATCTCTTTAAACCGCGCCCCTTAAGG 5'<br>RexT
S9	32P- ExoA P- Exo17<br>5'GTGGCGCGGAGACTTAGAGAA TTGGCGCGGGGAATTCC<br>CACCGCGCCTCTGAATCTCTTTAAACCGCGCCCCTTAAGG 5'<br>RexT
S10	32P- ExoA P- Exo18<br>5'GTGGCGCGGAGACTTAGAGAA TTTGGCGCGGGGAATTCC<br>CACCGCGCCTCTGAATCTCTTTAAACCGCGCCCCTTAAGG 5'<br>RexT

S11	32P- ExoA Exo19<br>5′GTGGCGCGGAGACTTAGAGAA ATTTGGCGCGGGGAATTCC<br>CACCGCGCCTCTGAATCTCTT-TAAACCGCGCCCCTTAAGG 5′<br>RexT
S12	32P- ExoA P- P- Exo19<br>5′GTGGCGCGGAGACTTAGAGAA ATTTGGCGCGGGGAATTCC<br>CACCGCGCCTCTGAATCTCTT-TAAACCGCGCCCCTTAAGG 5′<br>RexT
S13	32P- ExoA P- Exo19<br>5′GTGGCGCGGAGACTTAGAGAA ATTTGGCGCGGGGAATTCC<br>CACCGCGCCTCTGAATCTCTT-TAAACCGCGCCCCTTAAGG 5′<br>RexT
S14	32P- Exo15 P- Exo3′A-12-25<br>5′GTGGCGCGGAGACTT AGAGAAATTTGGCACGGGGAATTCC<br>CACCGCGCCTCTGAA-TCTCTTTAAACCGTGCCCCTTAAGG 5′<br>Rex12T
S15	32P- Exo15 P- Exo19<br>5′GTGGCGCGGAGACTT ATTTGGCGCGGGGAATTCC<br>CGCCTCTGAATCTCTTTAAACCGCGCCCCTTAAGG 5′<br>RexT-35
S16	P- ExoA P- Exo19<br>5′GTGGCGCGGAGACTTAGAGAA ATTTGGCGCGGGGAATTCC<br>CACCGCGCCTCTGAATCTCTT-TAAACCGTGCCCCTTAAGG 5′<br>-P Rex12T 32P
S17	P- Exo15 P- Exo3′A-12-25<br>5′GTGGCGCGGAGACTT AGAGAAATTTGGCACGGGGAATTCC<br>CACCGCGCCTCTGAA-TCTCTTTAAACCGTGCCCCTTAAGG 5′<br>P Rex12T 32P
S18	32P- 10-RT P- T19-RT<br>5′TGACTGCATA TGCATGTAGACGATGTGCAT<br>ACTGACGTAT-ACGTACATCTGCTACACGTA 5′<br>RT-A
S19	32P- 7 13db P- 18<br>5′GTCATTCGCTGTGCCCTCAA CGAATTCACAAGCCTAGA<br>3′CGACACGGGAGTTGGCTTAAGTGTTCGGATCT HEG linker<br>t t t 32
S20	32P-t 10db t P-t 22<br>5′GCTGTGCCCT CAACCGAATTCACAAGCCTAGA<br>3′CGACACGGGA-GTTGGCTTAAGTGTTCGGATCT HEG linker<br>t t t 32

[a]The symbol “t” designates modified nucleotide containing a thiophosphate group; “HEG linker” denotes hexaethyleneglycol linker [(CH2-CH2-O)6]; “Dbait” designates a long single-stranded hairpin

oligonucleotide in which complementary parts of DNA tethered with hexaethyleneglycol linker; "P" designates a phosphate group, "^{32}P" designates [γ-^{32}P]-ATP labeled oligonucleotide termini; "dAM^{32}P" designates [α-^{32}P]-3'-dATP (cordycepin 5'-triphosphate) labeled oligonucleotide termini; following abbreviations "ExoA", "Exo20", "Exo15", "10RT", "T19RT", "10db", "7-13db", "Exo19", "Exo18", "Exo17", "Exo14", "Exo3'A-12-25", "RexT", "Rex12T", "RexT-35", "RT-A" "50-db", "54-db" designate the single-stranded oligonucleotide fragments used to construct DNA structures. In DNA structure referred as "S19" the 7 nt 5' single-stranded overhang highlighted in red.

Supplementary Material Figures S1-S4.

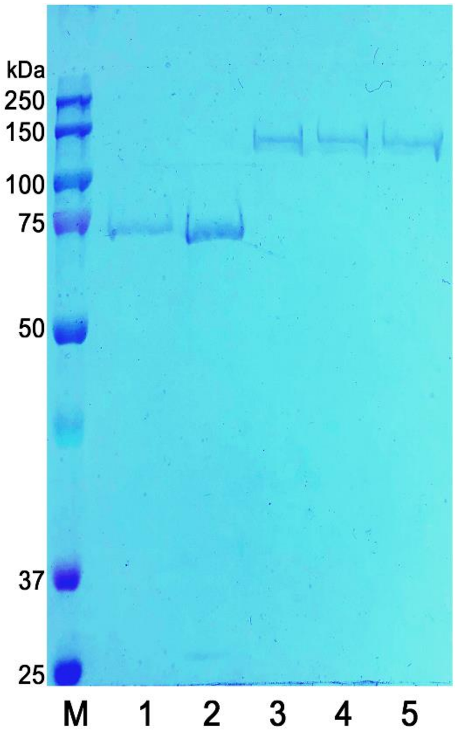

Supplementary Figure S1. SDS-PAGE analysis of the purified wild type and mutant Arabidopsis atPARP2 and atPARP1 proteins. Lane M, protein size markers; lane 1, 1 µg atPARP2-WT; lane 2, 1 µg atPARP2-E614K mutant; lane 3, 1 µg atPARP1-WT; lane 4, 1 µg atPARP1-E960K mutant; lane 5, 1 µg atPARP1 E960Q mutant. For details, see Materials and Methods.

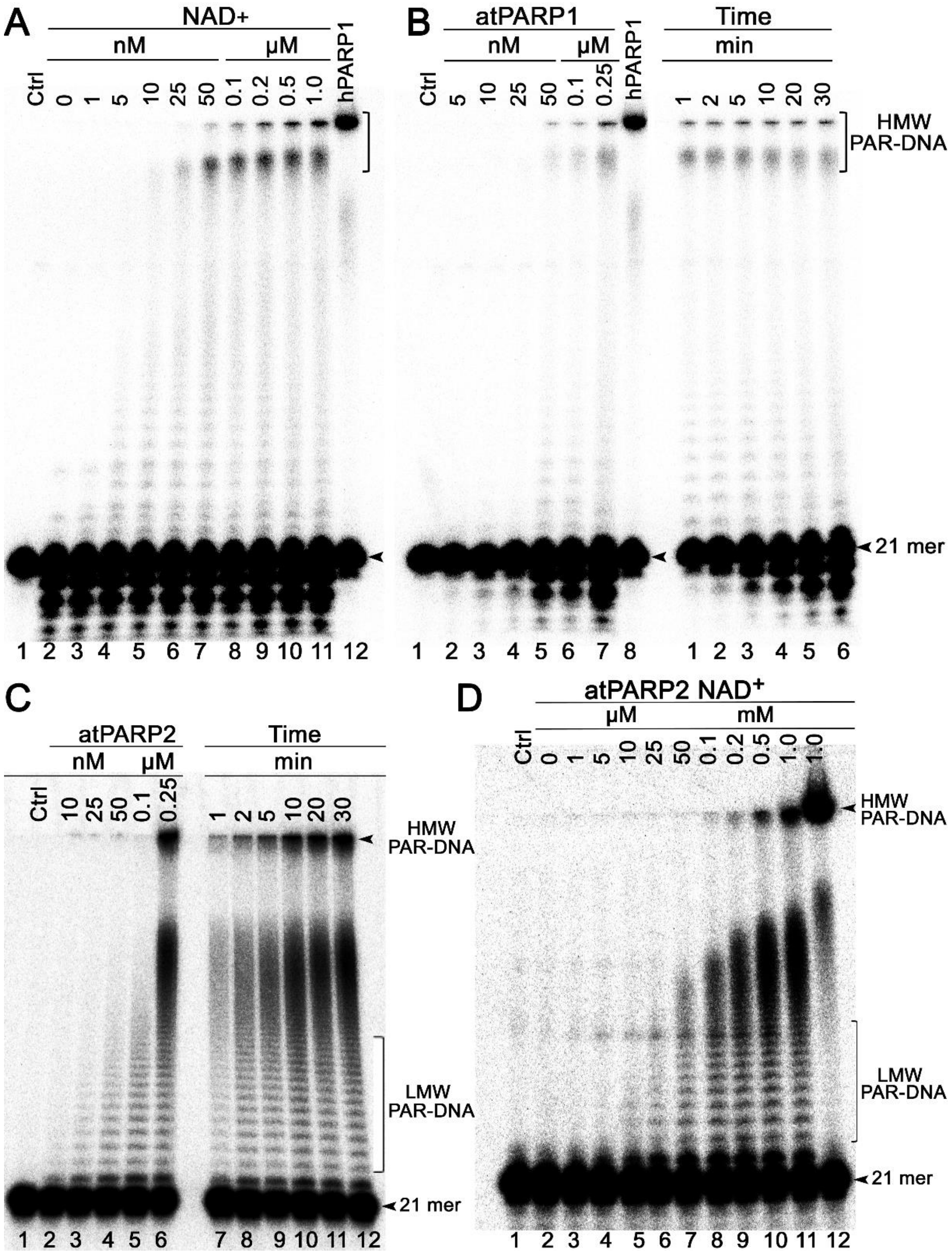

Supplementary Figure S2. The atPARP1- and atPARP2-catalysed DNA PARylation in the presence of varying concentrations of NAD^+, protein and incubation time. (**A**) Protein concentration and time dependence of atPARP1-catalysed DNA PARylation. (**B**) NAD^+ concentration dependence of atPARP1-catalysed DNA PARylation. (**C**) Protein concentration and time dependence of atPARP2-catalysed DNA PARylation. (**D**) NAD^+ concentration dependence of atPARP2-catalysed DNA PARylation.

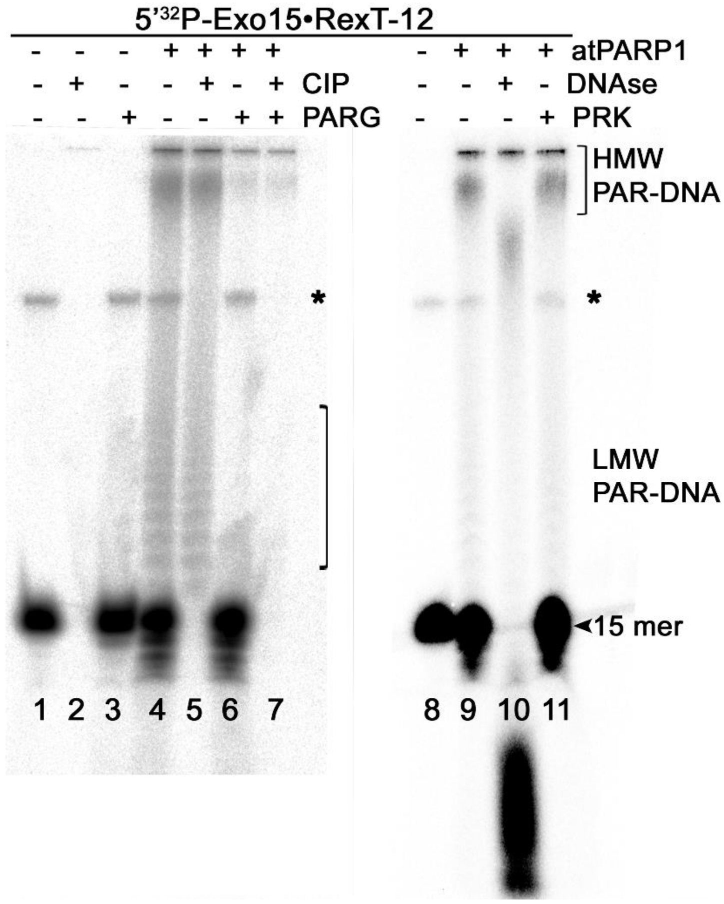

Supplementary Figure S3. Denaturing PAGE analysis of the products of digestion of PAR-DNA adducts by various enzymes. To generate PAR-DNA products 20 nM 5'-[^{32}P]-labelled Exo15•Rex12T^{rec} duplex was incubated with 250 nM atPARP1 in the presence of 1mM NAD^{+} for 30min at 37°C. After incubation, the reaction mixtures were heated for 20 min at 80°C and then incubated either in the presence of 50 pg•µL^{-1} PARG (in ADPR buffer) for 60 min at 37°C, or 10 U CIP (in CIP buffer) or 10.5 U DNAse I (in buffer with 0.5 mM $CaCl_2$) for 30 min at 37°C or 50 µg•mL^{-1} proteinase K for 30 min at 50°C in the presence of 0.1% SDS. Arrows indicate HMW and LMW PAR–DNA products and free 15 mer oligonucleotide. Asterisk indicates a nonspecific ligation product produced by *E. coli* NAD^{+}-dependent DNA ligase A. For more details, see Materials and Methods.

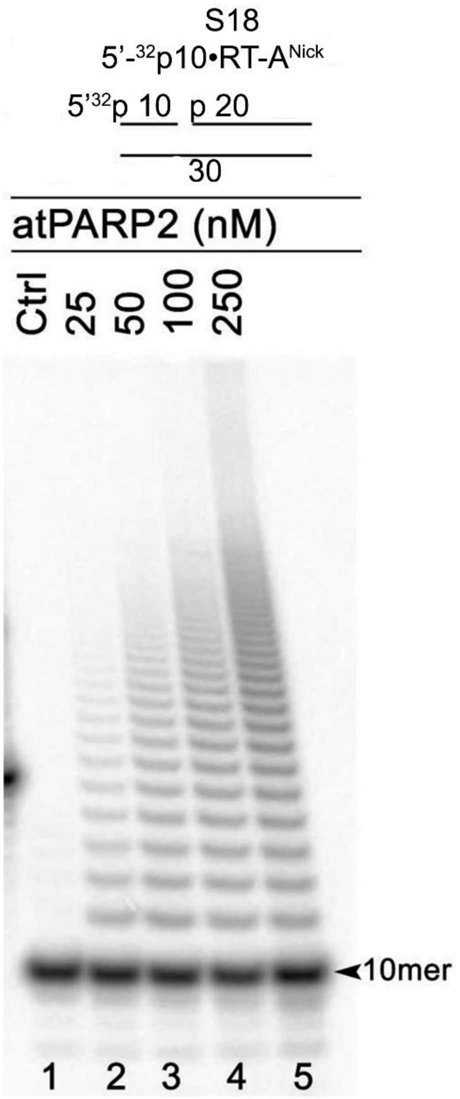

Supplementary Figure S4. Formation of the PAR-DNA products by atPARP2 for MALDI-TOF MS analysis. 25-250 nM atPARP2 was incubated with 20 nM 5'-[^{32}P]-labelled p10•RT-A^{Nick} oligonucleotide duplex (also referred as S18) in the presence of 1 mM NAD^{+} for 30 min at 37°C. The reaction products were analyzed by denaturing PAGE. Arrow indicates free 10 mer oligonucleotide. For more details, see Materials and Methods.

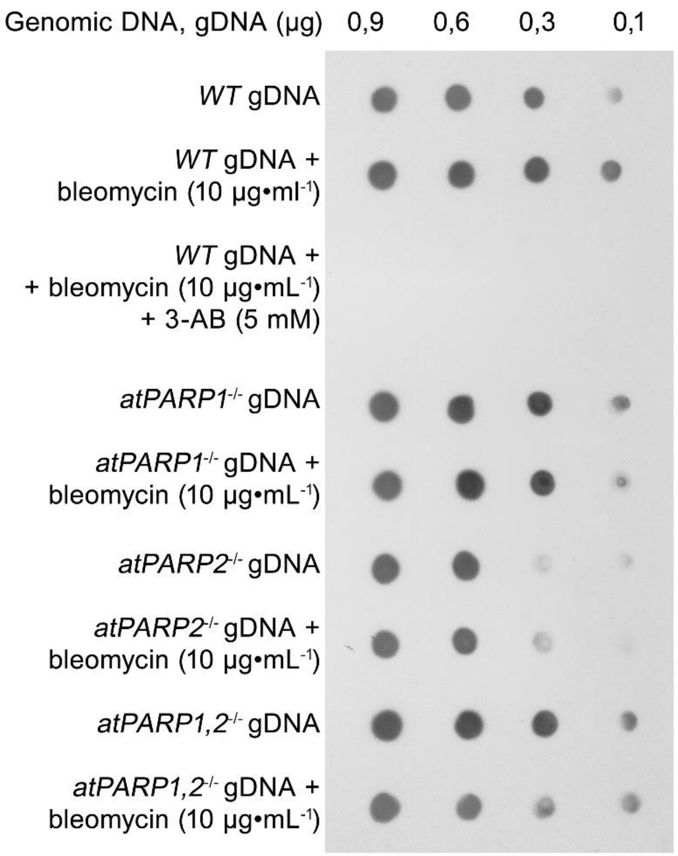

Supplementary Figure S5. Detection of PAR-DNA adducts in gDNA extracted from 14-days-old seedlings grown under either normal conditions or genotoxic stress. Different quantities of gDNA in TE buffer (10 mM Tris-Cl pH 8.0, 1 mM EDTA) were spotted onto a nylon membrane, followed by the pan-ADP-ribose reagent (MABE1016) dot blotting. For more details, see Materials and Methods.
